# Predisposition to hematopoietic malignancies by deleterious germline *CHEK2* variants

**DOI:** 10.1038/s41375-025-02635-1

**Published:** 2025-05-07

**Authors:** Ryan J. Stubbins, Stephen Arnovitz, Jennie Vagher, Anase Asom, Melody Perpich, Madeline Pies, Imo J. Akpan, Edward Chew, Joshua Bridgers, Aly Karsan, Courtnee Rodgers, Ashwin Koppayi, Hatice Basdag, Michael W. Drazer, Soma Das, Jason Cheng, Afaf E. G. Osman, Lucy A. Godley

**Affiliations:** 1 Leukemia/BMT Program of BC, BC Cancer, 2775 Laurel Street, V5Z 1M9 Vancouver, BC Canada; 2 Division of Hematology, Department of Medicine, University of British Columbia, 2775 Laurel Street, V5Z 1M9 Vancouver, BC Canada; 3 Section of Hematology Oncology, Department of Medicine, The University of Chicago, 5841 S. Maryland Ave., MC 2115, 60637 Chicago, IL USA; 4 Division of Hematology and Hematologic Malignancies, Department of Medicine, The University of Utah, 2000 Circle of Hope Dr, 84112 Salt Lake City, UT USA; 5 University of Chicago Pritzker School of Medicine 924 E 57th St #104, 60637 Chicago, IL USA; 6 Division of Hematology/Oncology, Department of Medicine, Columbia University Irving Medical Center, 161 Ft. Washington Ave, 10032 New York, NY USA; 7 Department of Diagnostic Hematology, The Royal Melbourne Hospital, 300 Grattan Street, Parkville, Victoria, 3052 Australia; 8 Laboratory Hematology, Austin Health, Studley Road, Heidelberg, Victoria, Australia; 9 Michael Smith Genome Sciences Centre, BC Cancer, 675 West 10th Avenue, Vancouver, BC V5Z 1L3 Canada; 10 Robert H. Lurie Comprehensive Cancer Center, Division of Hematology/Oncology, Northwestern University, 303 E. Superior St, 60611 Chicago, IL USA; 11 Department of Human Genetics, The University of Chicago, 5841 S. Maryland Ave., MC 0077, 60637 Chicago, IL USA; 12 Section of Hematopathology, Department of Pathology, The University of Chicago, 5841 S. Maryland Ave., MC 2115, 60637 Chicago, IL USA

**Keywords:** Cancer epigenetics, Cancer genetics

## Abstract

The role of germline *CHEK2* variants in hematopoietic malignancies (HMs) is poorly understood. We examined pathogenic/likely pathogenic (P/LP) *CHEK2* variants in patients with hereditary HMs (HHMs), a solid tumor risk cohort, public datasets, and a knock-in mouse model. In the HHM cohort, 57 probands had germline P/LP *CHEK2* variants, mostly p.I157T (53%, 30/57). Among *CHEK2* p.I157T carriers, 43% (19/44) had myeloid malignancies, 32% (14/44) had lymphoid malignancies, and 2% (1/44) had both. Among those with other germline P/LP *CHEK2* alleles, 36% (13/36) had myeloid malignancies, 28% (10/36) had lymphoid malignancies, and 6% (2/36) had both. *CHEK2* p.I157T was enriched in HM patients (OR 6.44, 95%CI 3.68–10.73, *P* < 0.001). In a solid tumor risk cohort, 36% (15/42) of *CHEK2* p.I157T patients had a HM family history. A genome wide association study showed enrichment of *CHEK2* loss-of-function variants with myeloid leukemia (*P* = 5.78e^−7^). In public acute myeloid leukemia (AML) datasets, 1% (16/1348) of patients had P/LP *CHEK2* variants. In a public myelodysplastic neoplasms (MDS) dataset, 2% (5/214) had P/LP *CHEK2* variants. *Chek2* p.I161T mice, homologous to human p.I157T, had worse survival as heterozygotes (*P* = 0.037) or homozygotes (*P* = 0.005), with fewer Lin-CD34+ and Lin-cKit+ cells. Our data suggest P/LP *CHEK2* variants are HHM risk alleles.

## Introduction

The *CHEK2* gene encodes the checkpoint kinase 2 (CHK2) protein, an integral effector kinase in the ATM-CHK2-TP53 DNA damage response (DDR) pathway [1, 2]. The Mre11 complex recognizes a DNA double-strand break (DSB) and activates ATM, which phosphorylates CHK2, promoting multimerization and autophosphorylation [3, 4]. Activated CHK2 phosphorylates downstream targets, including p53 and BRCA1, driving cell cycle slowing, apoptosis, and autophagy [5, 6]. Deleterious germline *CHEK2* variants are considered moderate penetrance risk alleles for breast [7–10] and prostate [7, 11] cancers, and possibly other solid organ malignancies [12–15]. These alleles are found at relatively high frequencies in some genetic subgroups, likely due to incomplete penetrance for cancers with onset beyond reproductive age, reducing selective pressure against them [16–18].

Small, single-center studies suggest that deleterious germline *CHEK2* variants may contribute to the development of non-Hodgkin lymphoma (NHL) [19, 20], myeloproliferative neoplasms (MPNs) [21, 22], and myelodysplastic neoplasms (MDS) [23, 24]. Genome wide association studies (GWAS) implicate deleterious germline *CHEK2* alleles as conferring increased risk for clonal hematopoiesis (CH) [25–28]. Reducing *CHEK2* expression by shRNA knockdown promotes the proliferation of Lin-CD34+ cells in long-term culture, a mechanism consistent with a CH phenotype [29]. In addition, deleterious *CHEK2* variants are common germline alleles detected in MDS or acute myeloid leukemia (AML) patients undergoing inherited cancer risk testing [30–32].

Lacking definitive studies describing the clinical and biological features of deleterious germline *CHEK2* variants in patients with hematopoietic malignancies (HMs), we examined the features of patients and populations with deleterious germline *CHEK2* variants and studied a knock-in mouse model of the most common such allele seen in HM patients, *CHEK2* p.I157T.

## Methods

### Ethics approval and consent to participate

All methods were performed in accordance with the relevant guidelines and regulations, as outlined below. A full list of primers, antibodies, and other reagents utilized is given in Supplemental Table 1, and further details of methods used are provided in the Supplemental Methods. Statistical comparisons were made using unpaired t-tests, and mouse survival was calculated by Kaplan-Meier with significance at *P* < 0.05, except where otherwise specified.

Written informed consent for all research participants was obtained at the University of Chicago (UC) under Institutional Review Board (IRB)-approved study 11-0014, and at the University of Utah under IRB-approved study 00046740.

Mice were maintained under UC IACUC-approved protocol 71370.

### Data collection and methods for clinical cohorts

Data were collected from HM patients with a known inherited cancer risk allele or a suspicion a hereditary hematologic malignancy (HHM) syndrome seen at UC from 2015 through 2022. Detailed inclusion criteria are specified in the Supplemental Methods. At the University of Utah, individuals with *CHEK2* p.I157T were identified through testing for solid tumor hereditary cancer risk. All variants were assessed by American College of Medical Genetics and Genomics/Association for Molecular Pathology (ACMG/AMP) guidelines (Supplemental Table 2). *CHEK2* variants were named according to MANE select transcript NM_007194.4. Population frequencies were obtained from gnomAD (v.4.1.0) [33]. Metadata for The Cancer Genome Atlas (TCGA) cohort was obtained from cBioPortal (https://www.cbioportal.org/) [34, 35].

### Genome-wide and phenome-wide association studies

Phenome-wide association study (PheWAS) for *CHEK2* (ENSG00000183765) with the predicted loss of function (pLoF) burden set and GWAS data for ICD-10 code C92 Myeloid leukaemia were obtained from the UK Biobank (UKBB) through the genebass portal (https://app.genebass.org/, GRCh38, 0.13.0-43c83cc-202402232123) with *P*-values expressed as optimized nonburden sequence kernel association tests (SKAT-O) [36–38].

### Identification of *CHEK2* variants in public AML datasets

Public RNA-seq data from BEAT AML, Leucegene, and AML PMP consortia were obtained for patients diagnosed with AML or high-grade myeloid neoplasm [39–41]. Public RNA-seq data from bone marrow mononuclear or CD34+ cells from MDS patients were obtained from EGAS00001002346 [42]. *CHEK2* variants were called as described in Supplemental Methods. Somatic variants and translocations were obtained from the associated metadata. Recurrent fusion events were defined as per European LeukemiaNet (ELN) 2022 definitions [43].

### Generation and characterization of a Chek2 mouse model

The *Chek2* p.I161T mouse was generated as a constitutional knock-in allele with T8677C/p.I161T in C57BL/6 mice. Mice were followed by twice weekly health checks until 24 months or a humane endpoint. The hematopoietic stem and progenitor cell (HSPC) compartment was examined by flow cytometry following column-based lineage depletion (Miltenyi Biotec). For bulk RNA-seq, Lin-CD34+ cells were flow sorted directly into RLT+ lysis buffer and sequenced on an Illumina NovaSeq PE100 S1 flowcell, followed by transcript quantitation with Salmon (v.1.9.0), and analyzed with DEseq2 (v.1.32.0) and GSEA (v.4.2.2) with the mSigDB m2.all v.2023 gene set [44]. Clonal hematopoiesis was assessed by somatic variant identification on DNA derived from PB collected from 8-9 month-old mice. Whole exome sequencing (WES) with target coverage of 150X was performed on the Illumina NovaSeq X plus with a S4 flowcell and 100 bp paired end reads and analyzed per the genome analysis toolkit (GATK) best practices workflow [45]. Mouse necropsies were performed on all deceased mice, including gross examination, complete blood cell count (CBC), blood smears, tissue sections, immunohistochemistry (IHC), and multicolor flow cytometry. B/T clonotype sequencing was performed with the NEBNext Mouse Immune Sequencing Kit on an Illumina MiSeq with 300 bp paired end reads and analyzed with MiXCR (v.4.4.2).

## Results

### *CHEK2* variants in a HHM testing cohort

**Fig. 1:**
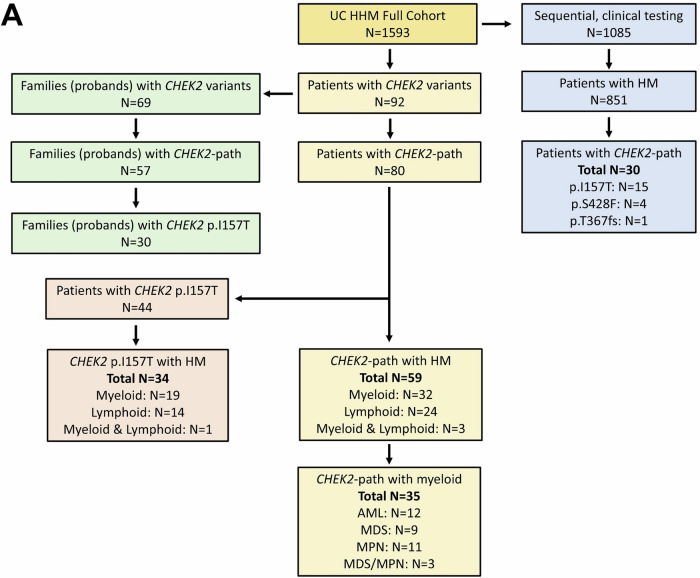
Breakdown of patients with *CHEK2* variants in the University of Chicago (UC) cohort. **A **A total of 80 patients from 57 families (unique probands) with pathogenic *CHEK2* variants (*CHEK2*-path) were identified from the UC HHM cohort. Amongst these 80 patients, 59 had a HM. In the subset of patients with the *CHEK2* p.I157T variant, 34 had a HM. In the subset of patients who were included in the allele burden calculation who had sequential, clinical testing (*N* = 1085), 851 had a HM and 30 of these had *CHEK2*-path, with the most common variant being *CHEK2* p.I157T (*N* = 15).

**Fig. 2:**
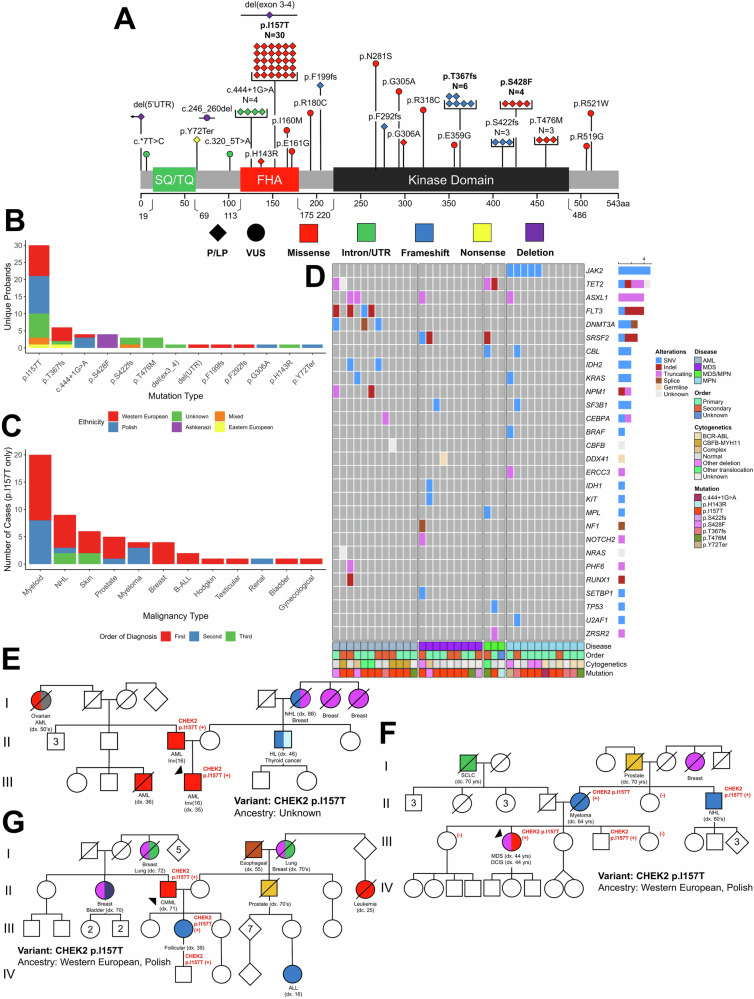
Germline *CHEK2* variants identified in a cohort of patients tested for HHM. **A** CHK2 protein schematic showing the locations of the encoded protein variants identified among 69 probands tested for HHMs. CHK2 protein domains are outlined: SQ/TQ (green bar), forkhead-associated (FHA) domain (red bar), and the kinase domain (black bar). Diamonds depict P/LP variants, and circles denote VUS. Red, missense variants; green, intronic and UTR variants; blue, frameshift variants; yellow, nonsense variants; and purple, deletion variants. *N* gives the number of independent probands/families with a particular allele. **B** The frequency with which individual alleles were identified amongst probands. Self-reported ethnicities were given by color: Western Europe, red; Polish, blue; Unknown, green; Ashkenazi, purple; Mixed, orange; Eastern Europe, yellow. **C** Distribution of HMs in those with the *CHEK2* p.I157T allele. Color indicates the order of the malignancy for those diagnosed with multiple cancers: first cancer, red; second cancer, blue; third cancer, green. **D** Cytogenetic and molecular profiles of the myeloid malignancies that developed in those with germline P/LP *CHEK2* variants. **E–G** Representative pedigrees from probands with P/LP germline *CHEK2* variants. The proband is indicated by the triangle. The germline *CHEK2* variant is indicated for those family members who were genotyped. Cancer diagnoses are given below each pedigree member along with the age at diagnosis (dx.) or death (dc.) when known. Circles indicate women, and squares, men. The generation number is given to the left in Roman numerals. (ns not significant. * *P* < 0.05, ** *P* < 0.01, *** *P* < 0.001).

Within a total of 1593 research participants in the UC HHM cohort [46], we identified 92 patients with *CHEK2* variants from 69 unique families (probands) (Fig. 1). Among all patients, including probands and family members, 87% (80/92) had pathogenic (P) or likely pathogenic (LP) germline *CHEK2* variants (*CHEK2*-path) and 13% (12/92) had variants of uncertain significance (VUS). Among *CHEK2* variants observed in probands, 83% (57/69) were *CHEK2*-path and 17% (12/69) were VUS; benign or likely benign variants were not reported (Supplemental Table 3). The most common *CHEK2* variant in the 57 probands with *CHEK2*-path variants was p.I157T (53%, 30/57) followed by p.T367fs (11%, 6/57), and p.S428F (7%, 4/57) (Fig. 2A). The most common *CHEK2*-path variant type was missense (68%, 39/57), followed by frameshift (19%, 11/57), and the most affected *CHEK2* protein region was the forkhead-associated (FHA) domain (63%, 36/57) followed by the kinase domain (32%, 18/57). The ethnic distribution within this cohort was predominantly European, and the most common specific ancestry for the p.I157T allele was Polish (37%, 11/30) and for p.S428F was Ashkenazi Jewish (100%, 4/4) (Fig. 2B), likely reflecting the underlying population within the greater Chicagoland area.

Among all probands and family members (patients) with *CHEK2*-path, 74% (59/80) had a diagnosed HM; 54% (32/59) had a myeloid malignancy, 41% (24/59) had a lymphoid malignancy, and 5% (3/59) had both myeloid and lymphoid malignancies. These myeloid malignancies occurred as second malignancies in 34% (12/35) of patients. In the subset of patients with *CHEK2* p.I157T (55%, 44/80), 77% (34/44) had a HM; 56% (19/34) had a myeloid malignancy, 41% (14/34) had a lymphoid malignancy, and 3% (1/34) had both myeloid and lymphoid malignancies (Fig. 2C). In the *CHEK2* p.I157T subset, (14/44) had ≥2 malignancies diagnosed over their respective lifetimes, versus 39% (14/36) for other P/LP *CHEK2* variants (*P* = 0.638, Fisher’s exact), and 17% (2/12) for *CHEK2* VUS (*P* = 0.475, Fisher’s exact). Less than half of the myeloid malignancies in *CHEK2* p.I157T patients were second malignancies (42%, 8/19). In the *CHEK2* p.I157T group, the frequency of breast cancer was 9% (4/44) and prostate cancer 11% (5/44). For other P/LP *CHEK2* variants, the frequency of breast cancer was 21% (9/36) and prostate cancer 5% (2/36).

Among all diagnosed myeloid malignancies in *CHEK2*-path patients (*N* = 35), 34% (12/35) had AML, 26% (9/35) had MDS, 31% (11/35) had MPN, and 9% (3/35) had MDS/MPN (Fig. 2D). In AML patients with *CHEK2*-path, 25% (4/12) had CBFB-MYH11 [inv(16)(p13.1q22.1)] rearrangement and 42% (5/12) had a normal karyotype. Somatic variants were similar to those seen in de novo AML. When comparing *CHEK2*-path AML patient cytogenetics with the distribution observed in unselected, de novo AML patients from the TCGA AML dataset, core binding factor (CBF) rearrangements [*CBFB-MYH11* or *RUNX1-RUNX1T1*] were more common in patients with *CHEK2*-path variants (25%, 4/12) versus TCGA de novo AML (10%, 19/200; *P* = 0.029, Fisher’s exact; Supplemental Fig. 1A). MDS/MPN was seen in three patients, all of whom had chronic myelomonocytic leukemia (CMML). Among MPN patients, 36% (4/11) had *BCR-ABL* fusions, and 27% (3/11) had myelofibrosis. Representative pedigrees of *CHEK2* p.I157T families with AML and inv(16)/*CBFB-MYH11* are shown (Fig. 2E); and with both myeloid and lymphoid malignancies (Fig. 2F, G). Treatment courses for individuals with germline *CHEK2* p.I157T and myeloid (Supplemental Fig. 2A) or lymphoid (Supplemental Fig. 2B) malignancies are also demonstrated. Among *CHEK2* p.I157T patients with myeloid malignancies, 40% (8/20) had a previous solid organ malignancy; 15% (3/20) had surgical treatment only, and 25% (5/20) had chemotherapy or radiation, one of whom had radiation for an antecedent Hodgkin lymphoma. Among *CHEK2* p.I157T patients with lymphoid malignancies, 33% (5/15) had a previous solid organ malignancy; 27% (4/15) had surgical treatment only, and 7% (1/15) had prior chemotherapy treatment.

Within the subset of the sequential probands that received routine clinical testing and had a personal history of myeloid or lymphoid malignancy and were tested for a clinical indication of suspected HHM, the frequency of *CHEK2*-path variants was 3% (30/851). When compared to the allele frequencies within a control population database (gnomAD v4.1.0, Supplemental Table 4), we observed a significant enrichment of the p.I157T (OR 6.44, 95% CI 3.86-10.73, *P* < 0.001) and p.S428F (OR 17.16, 95% CI 6.40-46.03, *P* < 0.001) alleles in patients with HMs, although the p.T367fs (OR 0.68, 0.10–4.87, *P* = 0.704) allele was not significant (Table 1).

**Table 1:** Allele burden calculation for sequential probands with hematopoietic malignancy receiving clinical germline testing versus control allele frequencies from gnomAD population database.

Sequential probands with hematopoietic malignancy and clinical germline testing			Control population (gnomAD v4.1.0)		Hematopoietic Malignancy vs. gnomAD	Sig.
Variant	Proportion with variant	Variant Frequency	Allele Number (heterozygous)	Allele Frequency	OR [95% Cl]	*P*
p.I157T	15 variants	0.01763	4,421 variants	0.00274	6.44 [3.86–10.73]	<0.001
(c.470 T > C)	851 tests		1,614,104 alleles			
p.S428F	4 variants	0.00470	441 variants	0.00027	17.16 [6.40–46.03]	*<*0.001
(c.1283 C > T)	851 tests		1,610,148 alleles			
p.T367fs	1 variants	0.00118	262 variants	0.00172	0.68 [0.10–4.87]	0.704
(c.1100delC)	851 tests		152,324 alleles			
Total *CHEK2*	30 P/LP variants	0.03525				
	851 total tests					

### Characteristics of patients with CHEK2 p.I157T in a solid tumor risk cohort

**Fig. 3:**
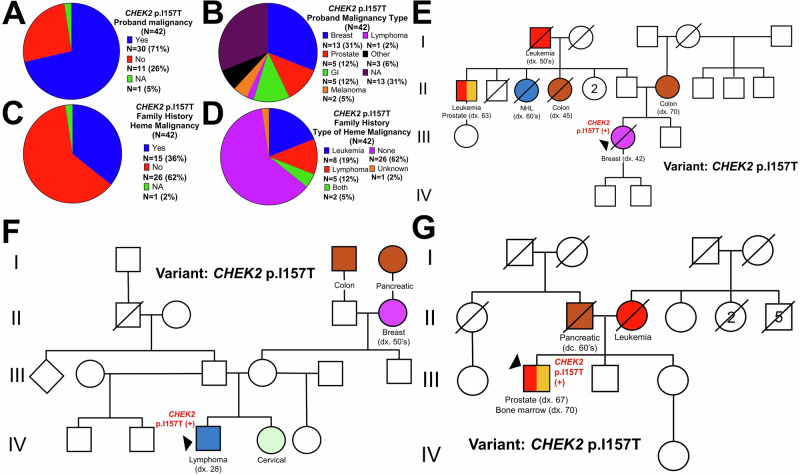
HM phenotype in patients with P/LP germline *CHEK2* variants tested for solid tumor cancer risk. Characteristics of patients tested for solid tumor risk indications at the University of Utah who have the *CHEK2* p.I157T variant. **A** Personal history of cancer for those with the *CHEK2* p.I157T allele. **B** Primary malignancy type for those with the *CHEK2* p.I157T allele who were diagnosed with cancer. **C** Family HM history for those with the *CHEK2* p.I157T allele. **D** Type of HM within the family for those with the *CHEK2* p.I157T allele. **E–G** Representative pedigrees of patients with the germline *CHEK2* p.I157T allele from the University of Utah cohort. The proband is indicated by the triangle. The germline *CHEK2* variant is indicated for those family members who were genotyped. Cancer diagnoses are given below each pedigree member along with the age at diagnosis (dx.) or deceased (dc.) when known. Circles indicate women, and squares, men. The generation number is given to the left in Roman numerals.

We identified 42 probands with *CHEK2* p.I157T at the University of Utah who underwent hereditary testing after referral for solid tumor hereditary cancer risk assessment. Among 42 probands, 71% (30/42) had a personal history of malignancy (Fig. 3A): primarily breast (31%, 13/42) and prostate (12%, 12/42) cancers, with one patient (2%, 1/42) having lymphoma (Fig. 3B). However, a total of 36% (15/42) of these probands reported a family history of HM (Fig. 3C), most commonly leukemia (19%, 8/42) followed by lymphoma (12%, 5/42), with two family histories containing both (5%, 2/42) (Fig. 3D). Representative pedigrees demonstrating diagnoses of both leukemias and lymphomas (Fig. 3E) and both solid and HMs (Fig. 3F, G) are shown.

### Phenome-wide and genome-wide association studies

**Fig. 4:**
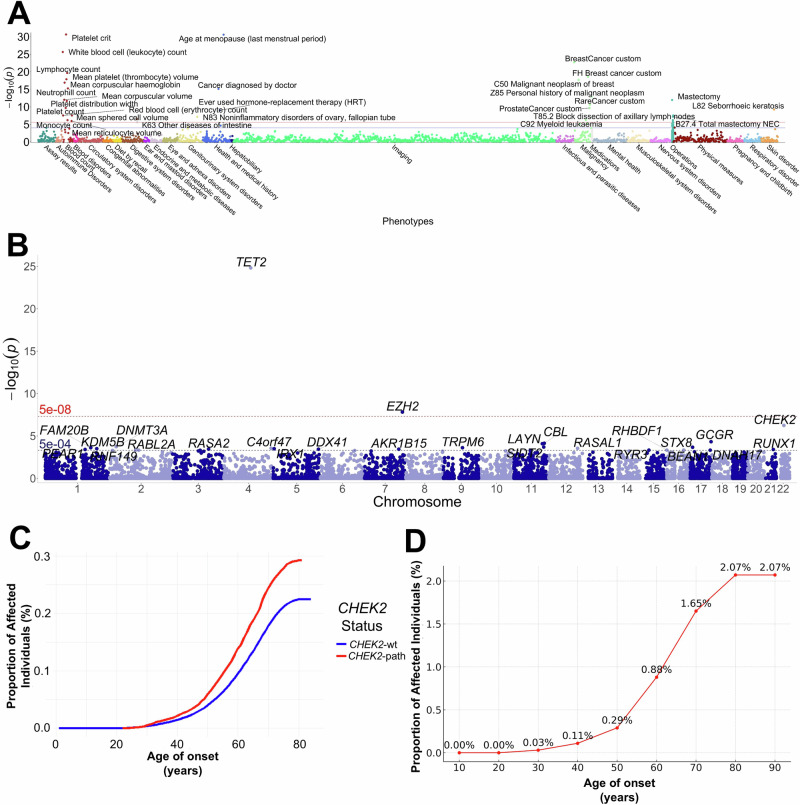
Phenome-wide association study (PheWAS) for *CHEK2* predicted loss of function (pLoF) variants and genome-wide association study (GWAS) for myeloid malignancy. **A** Phenome wide association study (PheWAS) for *CHEK2* predicted loss of function (pLoF) variants from the UK Biobank (UKBB). Hematologic phenome terms are indicated by dark red circles and malignancy diagnoses by light green circles. **B** Genome-wide association study (GWAS) from the UK Biobank for ICD-10 code C92 myeloid malignancy from the UK Biobank, significance lines indicated at -log_10_(5e^-08^) and -log_10_(5e^-04^) levels. **C** Cumulative proportion of participants from UKBB with malignant cancer who are *CHEK2*-wild type (wt) or carry pathogenic/likely pathogenic variants (*CHEK2*-path) by age of onset. **D** Cumulative proportion of *CHEK2*-path UKBB participants with HM diagnosis by age of onset.

Within the UKBB, we identified 4529 predicted loss of function (pLoF) gene burden associations for *CHEK2*-path variants by PheWAS (Supplemental Table 5). Among 36 phenotype terms significant at a SKAT-O *P*-value of <2.5e^−6^, 36% (13/36) were associated with blood count abnormalities (Fig. 4A). The top phenotype term was platelet crit (*P* = 2.33e^−31^, β = 0.008975), followed by age at menopause (*P* = 2.62e^−31^, β = 0.019220), and white blood cell (WBC) count (*P* = 2.30e^−26^, β = 0.008718). The two specific ICD-10 malignancy codes observed were C50 Malignant Neoplasm of Breast (*P* = 2.16e^−18^, β = 0.042475) and C92 Myeloid leukaemia (*P* = 5.78e^−7^, β = 0.092176). Within the UKBB, a GWAS for ICD-10 code C92 Myeloid leukaemia showed the strongest association with pLoF variants in the genes *TET2* (*P* = 1.53e^−25^, β = 0.176629), *EZH2* (*P* = 1.53e^−8^, β = 0.430923), and *CHEK2* (*P* = 5.78e^−7^, β = 0.092176) (Supplemental Table 6). Other known myeloid leukemia predisposition genes, including *DDX41* (*P* = 3.68e^−4^, β = 0.115770) and *RUNX1* (*P* = 3.99e^−4^, β = 0.176099), were identified at a lower significance level than seen for *CHEK2* pLoF variants (Fig. 4B).

In the UKBB population, *CHEK2* was the most observed HM risk allele (0.77%, 3867/502,410) followed by *BRCA2* (0.30%, 1504/502,410), *ATM* (0.30%, 1485/502,410), *DDX41* (0.15%, 750/502,410), *BRCA1* (0.11%, 533/502,410), *TP53* (0.01%, 61/502,410), and *RUNX1* (0.00%, 1/502,410) (Supplemental Table 9). To assess an association between *CHEK2* pLoF variants and HMs, we performed univariate and multivariate logistic regression for sex, age, *CHEK2* status, and smoking status after filtering out UKBB participants with non-*CHEK2* likely germline cancer risk alleles (Supplemental Fig. 3). For all HMs, *CHEK2* status was significant on multivariate analysis (OR 1.91, 95% CI 1.52–2.37, *P* < 0.001; Supplemental Fig. 4A). For myeloid malignancies, *CHEK2* status was also significant on multivariate analysis (OR 2.34, 95% CI 1.31–3.82, *P* = 0.002) (Supplemental Fig. 4B). *CHEK2* status was also significant for all malignant cancers, solid tumors, lymphoid malignancies, breast malignancy, prostate malignancy, and thyroid malignancy, but was not significant for lung or colon malignancy (Supplemental Fig. 5; Supplemental Table 10). In cancer patients with likely germline *CHEK2*^*LoF*^ variants, the penetrance for any malignancy was 29.27% by age 90 (Fig. 4C). The penetrance by age 90 for solid tumors was 25.97% (Supplemental Fig. 6A); and for all HMs, was 2.07% (Fig. 4D). Among UKBB participants with AML, 58% (11/19) were de novo, and 42% (8/19) were secondary AML (Supplemental Fig. 6B).

### Identification of *CHEK2* variants in public MDS/AML datasets

**Fig. 5:**
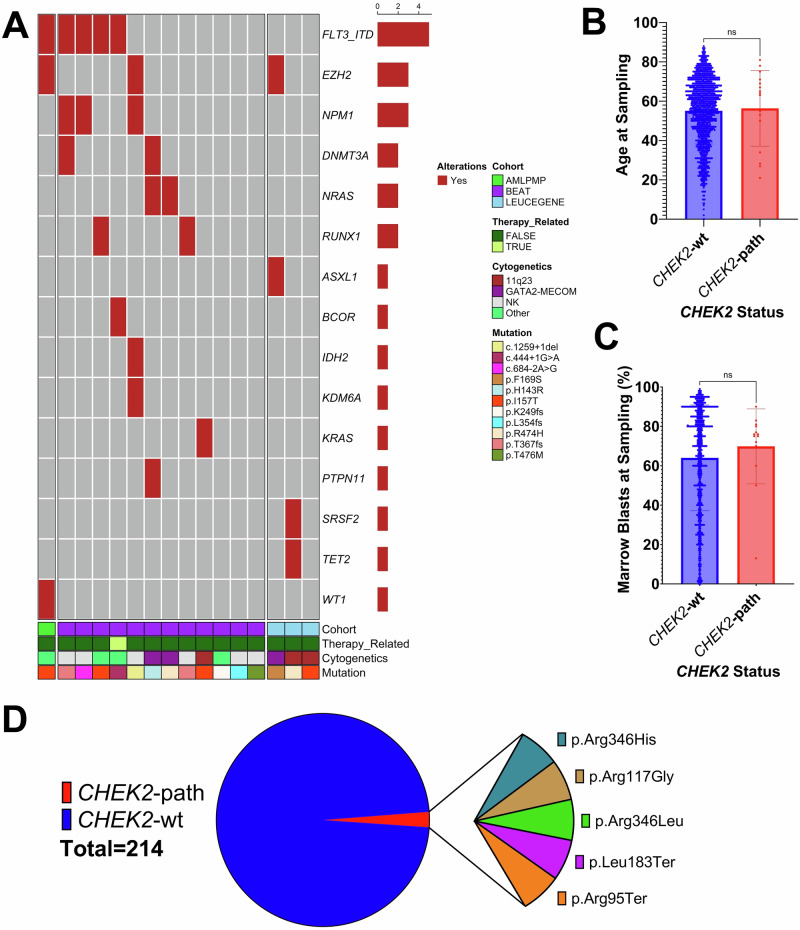
Characteristics of MDS and AML patients with P/LP *CHEK2* variants called from public RNA-seq datasets. **A** Somatic mutational landscape and disease characteristics of patients with AML with identified *P/LP CHEK2* variants. **B** Median age at time of disease sample in AML patients who are *CHEK2*-wt (blue, left) vs. those with *CHEK2*-path variants (red, right). **C** Burden of bone marrow blasts at time of disease sample in AML patients who are *CHEK2*-wt (blue, left) vs. *CHEK2*-path (red, right). **D** Frequency of *CHEK2*-path variants in a cohort of MDS patients with bulk RNA-seq from bone marrow mononuclear cells or CD34+ cells. (ns not significant, * *P* < 0.05, ** *P* < 0.01, *** *P* < 0.001).

*CHEK2* variants were called from bulk RNA-seq reads in three large public AML datasets (BEAT AML, Leucegene, AML PMP) to ensure consistency among studies [39–41]. We identified P/LP *CHEK2* variants in 1.2% (16/1348) of AML patients in the combined dataset (Fig. 5A). The most common *CHEK2* variant was p.I157T in 25% (4/16) followed by p.T367fs in 13% (2/16). Only 6% (1/16) were identified as therapy-related myeloid neoplasms in the metadata. There was no difference in the mean age at sampling between those with P/LP *CHEK2* variants (*CHEK2*-path) (56.33 years) versus wild type (*CHEK2*-wt) (55.11 years) (*P* = 0.774) (Fig. 5B). There was no difference in the number of females with *CHEK2*-path (44%, 7/16) versus *CHEK2*-wt (44%, 563/1283) (*P* > 0.999). There was no difference in the mean WBC at sampling between *CHEK2*-path (37.38×10^9^) versus *CHEK2*-wt (49.04 × 10^9^) (*P* = 0.492) (Fig. 5C). We did not observe an enrichment for *CBF* fusions in *CHEK2*-path AML patients at 0% (0/16) versus *CHEK2*-wt at 9% (128/1348) (*P* = 1, Fisher’s exact). However, we did observe an enrichment for *GATA2*-*MECOM* fusions in *CHEK2*-path at 19% (3/16) versus *CHEK2*-wt at 1% (17/1348) (*P* = 0.001, Fisher’s exact) and non-*MLLT3 KMT2A* rearrangements at 19% (3/16) in *CHEK2*-path versus *CHEK2-*wt at 2% (31/1348) (*P* = 0.006, Fisher’s exact) (Supplemental Fig. 1B). We then examined the frequency of *CHEK2*-path variants in a genetically distinct cohort of MDS patients with bulk RNA-seq from bone marrow mononuclear or CD34+ cells [42]. We identified *CHEK2*-path variants in 2% (5/214) of MDS patients in this dataset, and did not observe *CHEK2* p.I157T, consistent with the expected population distribution of this allele (Fig. 5D).

### Generation and characterization of a Chek2 p.I161T mouse model

The human and mouse CHK2 proteins share 83.5% amino acid identity with the mouse CHK2 I161 residue corresponding to the human CHK2 I157 residue (Supplemental Fig. 7). We generated a p.I161T C57BL/6 J knock-in mouse, and confirmed the presence of this allele in tail, bone marrow, and spleen; expression of both wild-type and p.I161T alleles was confirmed in the spleen of *Chek2*^*p.I161T/w*t^ mice (Supplemental Fig. 8). Both heterozygotes (*Chek2*^*p.I161T/w*t^) and homozygotes (*Chek2*^*p.I161T/p.I161T*^) were viable and fertile, without developmental defects. Comparable gene and protein expression of *Chek2* was identified in bone marrow and spleen for both heterozygotes and homozygotes with the *Chek2* p.I161T allele (Supplemental Fig. 9).

**Fig. 6:**
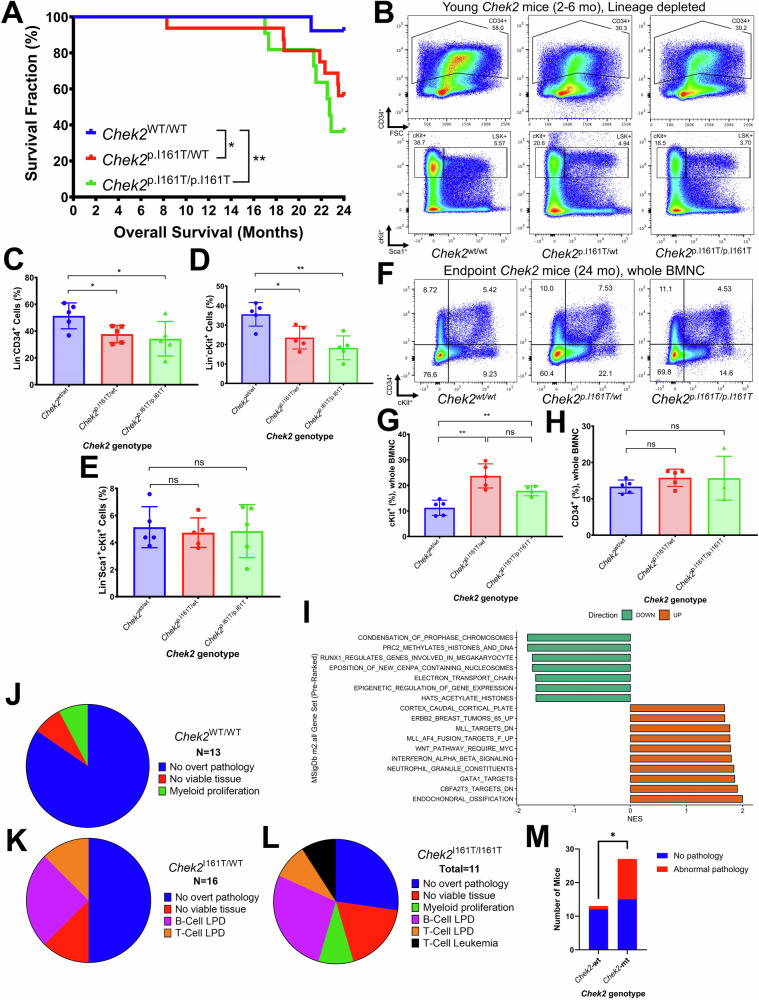
Hematopoietic stem and progenitor (HSPC) features of a *Chek2* p.I161T mouse model. **A** Survival in *Chek2*^*wt/wt*^ (blue, *N* = 13), *Chek2*^*p.I161T/wt*^ (red, *N* = 16), vs. *Chek2*^*p.I161T/p.I161T*^ (green, *N* = 11) mice. **B** Representative flow plots from lineage depleted marrow demonstrating the Lin-CD34+ and Lin-Sca1+cKit+ compartments in young (2-6 month) *Chek2* wt (*Chek2*^*wt/wt*^), heterozygous (*Chek2*^*p.I161T/wt*^), and homozygous (*Chek2*^*p.I161T/p.I161T*^) mice. **C** Comparison of Lin-CD34+ compartment in young *Chek2* mice. **D** Comparison of Lin-cKit+ compartment in young *Chek2* mice. **E** Comparison of the Lin-Sca1+cKit+ compartment in young *Chek2* mice. **F** Representative flow plots from whole bone marrow nucleated cells (BMNC) demonstrating the CD34+ and cKit+ in aged (24 month) *Chek2* wt (*Chek2*^*wt/wt*^), heterozygous (*Chek2*^*p.I161T/wt*^), and homozygous (*Chek2*^*p.I161T/p.I161T*^) mice without pathologic evidence of malignancy. **G** Comparison of cKit+ compartment in aged *Chek2* mice. **H** Comparison of CD34+ compartment in aged *Chek2* mice. **I** Pre-ranked gene set enrichment analysis (GSEA) for differentially expressed genes from Lin-CD34+ cells from young (aged 5-6 month) *Chek2* mice. Highlighted pathways are significant at a false discovery rate (FDR) of <0.25. Pathways from MSigDb m2.all gene set with some titles abbreviated. Full pathway titles and details are in Supplemental Table 7. **J–L** Summary of consensus diagnosis obtained by analysis of gross features, histology, IHC, and multicolor flow cytometry data from wt (*Chek2*^*wt/wt*^), heterozygous (*Chek2*^*p.I161T/wt*^), or homozygous (*Chek2*^*p.I161T/p.I161T*^) mice. **M** Comparison of mice with identified pathologic abnormalities at endpoint for *Chek2*-wt (left) vs. *Chek2*-mt (right), including heterozygous or homozygous mice. (ns, not significant, * *P* < 0.05, ** *P* < 0.01, *** *P* < 0.001).

Longitudinal characterization of *Chek2* p.I161T mice over 24 months demonstrated a progressive increase in WBC for both *Chek2*^*p.I161T/wt*^ (*P* < 0.001, ANOVA) and *Chek2*^*p.I161T/p.I161T*^ (*P* < 0.001, ANOVA) mice versus wt (Supplemental Fig. 10A). There was an increase in absolute neutrophil count (ANC) in *Chek2*^*p.I161T/p.I161T*^ (*P* < 0.001, ANOVA) mice, but not in *Chek2*^*p.I161T/wt*^ (*P* = 0.514, ANOVA) mice versus wt (Supplemental Fig. 10B). There was an increase in absolute lymphocyte count (ALC) for both *Chek2*^*p.I161T/wt*^ (*P* < 0.001, ANOVA) and *Chek2*^*p.I161T/p.I161T*^ (*P* < 0.001, ANOVA) mice versus wt (Supplemental Fig. 10C). At endpoint, although there was no difference in hemoglobin levels among *Chek2*^*p.I161T/wt*^ (*P* = 0.212), *Chek2*^*p.I161T/p.I161T*^ (*P* = 0.163) mice, and wt (Supplemental Fig. 10D), we observed a higher platelet count in *Chek2*^*p.I161T/wt*^ *(P* = 0.018) but not *Chek2*^*p.I161T/p.I161T*^ (*P* = 0.058) as compared to wt mice (Supplemental Fig. 10E). We observed an inferior overall survival for both *Chek2*^*p.I161T/wt*^ (*P* = 0.037) and *Chek2*^*p.I161T/p.I161T*^ (*P* = 0.005) compared to wt mice, although this difference only emerged after the mice had been aged for more than 18 months (Fig. 6A).

### Hematopoietic stem and progenitor cell (HSPC) defects and assessment for clonal hematopoiesis (CH) in the Chek2 p.I161T mouse model

To assess the effects of the *Chek2* p.I161T allele on the HSPC compartment, we stained lineage (Lin)-depleted BMNC from *Chek2* mice younger than six months with antibodies against Lin, CD34, cKit, and Sca-1 and performed flow cytometry (Fig. 6B). We observed a reduction in Lin-CD34+ cells in mice with *Chek2*^*p.I161T/wt*^ (*P* = 0.030) or *Chek2*^*p.I161T/p.I161T*^ (*P* = 0.045) versus wild-type, but no difference between *Chek2*^*p.I161T/wt*^ and *Chek2*^*p.I161T/p.I161T*^ (*P* = 0.604) mice (Fig. 6C). Similarly, we observed a reduction in Lin-cKit+ cells in *Chek2*^*p.I161T/wt*^ (*P* = 0.013) or *Chek2*^*p.I161T/p.I161T*^ (*P* = 0.002) versus wild-type mice, but saw no difference between *Chek2*^*p.I161T/wt*^ and *Chek2*^*p.I161T/p.I161T*^ (*P* = 0.204) mice (Fig. 6D). Interestingly, there was no difference in the Lin-Sca1+cKit+ (LSK) compartment between *Chek2*^*p.I161T/wt*^ (*P* = 0.640) or *Chek2*^*p.I161T/p.I161T*^ (*P* = 0.800) versus wild-type mice, nor between *Chek2*^*p.I161T/wt*^ and *Chek2*^*p.I161T/p.I161T*^ (*P* = 0.911) mice (Fig. 6E).

To assess these features, mice ~24 months of age were sacrificed without evidence of malignancy, and we examined the proportion of whole blood mononuclear cells (BMNC) that was CD34+ or cKit+ (Fig. 6F). We observed an increase in the cKit+ population in *Chek2*^*p.I161T/wt*^ (*P* = 0.001) and *Chek2*^*p.I161T/p.I161T*^ (*P* = 0.006) versus wild-type mice, with no difference between *Chek2*^*p.I161T/wt*^ and *Chek2*^*p.I161T/p.I161T*^ (*P* = 0.056) mice (Fig. 6G). We observed no difference in the CD34+ compartment among *Chek2*^*p.I161T/wt*^ (*P* = 0.106) and *Chek2*^*p.I161T/p.I161T*^ (*P* = 0.436) versus wild-type mice, with no difference between *Chek2*^*p.I161T/wt*^ and *Chek2*^*p.I161T/p.I161T*^ (*P* = 0.968) mice (Fig. 6H).

We assessed transcriptional differences and changes to underlying signaling pathways by performing bulk RNA-seq on sorted Lin-CD34 + BMNC from mice aged 5-6 months. We observed differential gene expression (DGE) between *Chek2-wt* and *Chek2-mutant (mt)* (combined heterozygotes and homozygotes), with 22 genes being up- or downregulated (log2FC ± 1.5, *P* < 0.05) (Supplemental Fig. 11A). We observed similar DGE in *Chek2*^*p.I161T/wt*^ and *Chek2*^*p.I161T/p.I161T*^ separately versus wt (Supplemental Fig. 11B, C). but saw minimal DGE between *Chek2*^*p.I161T/wt*^ and *Chek2*^*p.I161T/p.I161T*^ (Supplemental Fig. 11D). Unsupervised clustering grouped *Chek2*^*wt/wt*^ samples together, with *Chek2*^*p.I161T/wt*^ and *Chek2*^*p.I161T/p.I161T*^ samples clustering separately (Supplemental Fig. 11E).

We identified ten upregulated gene sets (Supplemental Table 7) and seven downregulated gene sets gene set enrichment analysis (GSEA) at a false discovery rate (FDR) < 0.25. (Fig. 6I) Several hematopoietic-specific transcription factor (TF) networks were upregulated, including CBFA2T3 targets (NES 1.9062, nominal *P* < 0.001, FDR q-value 0.0199), GATA1 targets (NES 1.8583, nominal *P* < 0.001, FDR *q*-value 0.0402), and MLL targets (NES 1.7717, nominal *P* = 0.002, FDR *q*-value 0.0864). Some pathways important for epigenetic regulation and differentiation were identified in the downregulated gene list, including PRC2 Methylates Histones and DNA (NES -1.8309, nominal *P* < 0.001, FDR *q*-value 0.0152) and Epigenetic Regulation of Gene Expression (NES -1.6826, nominal *P* < 0.001, FDR q-value 0.1711). Downregulated pathways not significant at FDR < 0.25 included DNA DSB Response (NES -1.4846, nominal *P* = 0.0119, FDR q-value 0.6892) and NHEJ (NES -1.5063, nominal *P* 0.0261, FDR q-value 0.6954) (Supplemental Table 7).

To assess for the presence of CH, we performed deep WES on *Chek2* mice less than ten months old. After variant calling and filtering (Supplemental Fig. 12), we did not identify differences in the number of somatic variants in *Chek2*^*p.I161T/wt*^ (*P* = 0.252) or *Chek2*^*p.I161T/p.I161T*^ (*P* = 0.552) versus *Chek2*^*wt/wt*^ mice, a difference in VAF, or allele type (Supplemental Fig. 13). There was also no difference in the top 100 somatic variants by *Chek2* genotype (Supplemental Fig. 14).

### The Chek2 p.I161T mouse model develops HMs

We comprehensively profiled aged *Chek2* mice that were sacrificed at humane endpoint or 24 months. Several mice demonstrated abnormal necropsy findings (Supplemental Table 8). For example, one mouse had a disorganized hypercellular marrow with megakaryocyte proliferation (Supplemental Fig. 15A) and abnormal circulating monocytoid cells (Supplemental Fig. 15B) and abnormal circulating lymphocytes in another mouse (Supplemental Fig. 15C). Immunohistochemical stains from three separate mice showed diffuse infiltration with PAX5+ cells in a mass (Supplemental Fig. 15D) without CD3+ infiltrate (Supplemental Fig. 15E), diffuse splenic infiltration with lymphocytes that was PAX5- (Supplemental Fig. 15F) and CD3+ (Supplemental Fig. 15G), and a mass with a lymphocytic infiltrate that was PAX5- (Supplemental Fig. 15H) and CD3+ (Supplemental Fig. 15I). Within the *Chek2*^*wt/wt*^ group, 85% (11/13) had no overt pathology, 8% (1/13) had no viable tissue, and 8% (1/13) had evidence of a myeloid proliferation (Fig. 6J). For the *Chek2*^*p.I161T/wt*^ group, 50% (8/16) had no overt pathology, 13% (2/16) had no viable tissue, 25% (4/16) had evidence of a B-cell lymphoproliferative disorder (LPD), and 13% (2/16) had evidence of a T-cell LPD (Fig. 6K). In the *Chek2*^*p.I161T/p.I161T*^ group, 27% (3/11) had no overt pathology, 18% (2/11) had no viable tissue, 9% (1/11) had a myeloid proliferation, 27% (3/11) had a B-cell LPD, and 18% (2/11) had evidence of a T-cell LPD or leukemia (Fig. 6L). More mice had pathologic abnormalities suggestive of a hematopoietic disorder in the *Chek2*-mt group versus *Chek2*-wt (*P* = 0.030, Fisher’s exact; Fig. 6M). There was no difference in the number of mice with abnormalities among males (50%, 7/14) versus females (38%, 5/13; *P* = 0.704).

Representative flow plots demonstrate: a CD3 + CD4 + T-cell leukemia in a *Chek2*^*p.I161T/p.I161T*^ mouse (Supplemental Fig. 16A), transplantable to a CD45.1 recipient (Supplemental Fig. 16B), and clonality demonstrated by the presence of a dominant clone by TCR sequencing (Supplemental Fig. 17A); a CD34 + CD11b + CD117+ proliferation in a *Chek2*^*p.I161T/p.I161T*^ mouse (Supplemental Fig. 16C); and a B-cell lymphoproliferative disease (LPD) in a *Chek2*^*p.I161T/wt*^ mouse (Supplemental Fig. 16D), with a dominant clone identified by BCR sequencing (Supplemental Fig. 17B).

## Discussion

Our findings support deleterious germline *CHEK2* variants, and specifically p.I157T, as being a predisposition gene for the development of myeloid and possibly lymphoid malignancies. We observed evidence of a HM phenotype in patients and families with P/LP *CHEK2* variants in a HHM cohort, as well as a separate validation cohort that was tested for solid tumor hereditary cancer risk. The presence of strong family histories of leukemia in individuals with deleterious germline *CHEK2* variants who were tested for inherited cancer risk based on solid tumor history suggests that it is the P/LP *CHEK2* variant driving the observed HM predisposition, rather than being a second order effect arising from the selected nature of our HHM cohort. We also observe that, on a population level, LoF *CHEK2* variants associate with hematopoietic abnormalities and myeloid leukemias; although *CHEK2* was below the conventional 5e^−8^ threshold for GWAS analysis, HMs are underrepresented in the UKBB, limiting sensitivity in this context. Interestingly, *CHEK2* arises as one of the genes most strongly associated with myeloid leukemias by GWAS, suggesting a link between P/LP *CHEK2* P/LP variants and myeloid leukemias, but not implying high penetrance. P/LP variants that are higher penetrance but rarer are likely underrepresented in the UK Biobank, which may explain why we observe *RUNX1* and *DDX41* at a lower significance level in this analysis. We were unable to assess formally for the penetrance of *CHEK2* P/LP variants on the HM phenotype in our cohort. We also observe that *CHEK2* P/LP variants do not seem to be highly specific to a specific disease subtype within the family of myeloid diseases, though most patients had MDS or AML. However, we do see that there is an enrichment in some recurrent translocation events; within the original HM cohort CBF rearrangements were enriched, but surprisingly, we did not observe this in the broader AML cohort from public datasets. Instead, we saw enrichment of both *GATA2*-*MECOM* and t(v;11q23.3)/*KMT2A* rearrangements. These findings suggest that *CHEK2* P/LP variants may be a proximal risk factor for the development of translocation events, perhaps related to defective homologous recombination pathways, rather than a specific HM disease state. This could also explain the spectrum of HMs we observed, as translocation events are not exclusive to a single HM subtype [47]. Our findings also suggest *CHEK2*-associated HHM syndromes may be relatively common, based on our population data and the frequency in AML datasets, consistent with previous studies [30–32]. Overall, in public datasets, we observed 1-2% of MDS/AML patients had P/LP *CHEK2* variants with a comparable age of onset to non-*CHEK2* mutated HM patients, with only 6% of these being clearly identified as therapy-related, and a possible enrichment for recurrent translocation events in P/LP *CHEK2* carriers with MDS/AML.

Our findings also suggest that *CHEK2* likely plays a role in the HSPC compartment. Although we saw Lin-CD34+ and Lin-cKit+ populations were reduced in young *Chek2* mice, the LSK compartment was unchanged, suggesting that the impact of *Chek2* p.I161T is likely manifested at the level of an oligopotent progenitor (e.g., common myeloid progenitor) rather than a long-term hematopoietic stem cell. Interestingly, in older mice, we observed a reversal in this phenomenon with increased cKit+ cells. There are many possible mechanisms for this, but evolving CH as a compensatory mechanism for a HSPC defect is possible, given a similar phenomenon is seen in patients with germline marrow failure syndromes such as *SAMD9/SAMD9L* [48]. Although we tested younger mice for the presence of CH by deep WES performed on PB, we did not observe the presence of CH, likely reflecting the young age of the mice; limitations of using PB in mice; and the lack of serial blood draws within this cohort. Many of the differentially enriched pathways in our GSEA relate to hematopoiesis-specific TFs, raising the possibility that CHK2 is either directly or indirectly regulating these TFs. *CHEK2* is known to interact with several proteins that affect leukemia-specific pathways, such as KMT2D and FOXM1, but this remains to be explored further [49]. These findings suggest *CHEK2* may be relevant to HSPC regulation. Our mouse model also demonstrates that mice carrying *Chek2* p.I161T in the heterozygous or homozygous state have worse survival than *Chek2* wild-type mice, and that this is likely related to HM development. Future studies will examine the role that cytotoxic and radiation exposures have on the development of HMs in *Chek2*-mutant mice as deeper understanding regarding the interactions among *CHEK2* mutational status, environmental exposures, and HM risk will be crucial. Future in vitro studies should also examine whether certain drugs that are particularly effective in cells with defects in homologous recombination DNA repair (e.g., PARP inhibitors) are effective in eliminating malignant, *CHEK2*-defective hematopoietic cells.

Limitations of our study include the selected nature of our HHM cohort, being mostly European-derived, and our inability to correct for differences in ethnic composition versus gnomAD. Future studies should examine *CHEK2* variants in diverse populations. Sample size limitations also prevented detailed assessment of all disease subtypes. The role for co-inherited modifiers of the *CHEK2* phenotype also could not be assessed in this study. We are also limited in knowing the germline status of *CHEK2* variants in public datasets, although somatic *CHEK2* variants are rare [31]. We were also limited in our mouse studies by the rarity of aged (> 18 mo) *Chek2* mice, resulting in a need to perform several experiments on younger mouse cohorts.

Our findings establish that *CHEK2* should be routinely tested in all patients being assessed for a HHM syndrome, and likely should be tested for in all patients with a myeloid malignancy. Our observed frequency of 1% in the AML cohort and 2% in the MDS cohort is probably an underestimate, given that any variant not producing a transcript would not have been identified. Identification of *CHEK2* variants in HM patients has important implications for counseling and cascade testing, selection of allogeneic hematopoietic stem cell transplant donors, and may inform future clinical trials of targeted treatment approaches for this genetically distinct patient group.

## Supplementary information

Supplemental Material

Supplemental Table 1

Supplemental Table 2

Supplemental Table 3

Supplemental Table 4

Supplemental Table 5

Supplemental Table 6

Supplemental Table 7

Supplemental Table 8

Supplemental Table 9

Supplemental Table 10

## Data Availability

Sequencing data are deposited in SRA (PRJNA1119484) and GEO (GSE268966).

## Code availability

Code used for UK Biobank data analysis can be found on the Godley Lab github site: https://github.com/LucyGodley/UKB_CHEK2.
