# Supplementary material for: Predisposition to hematopoietic malignancies by deleterious germline CHEK2 variants
Source: Leukemia. 2025 May 7;39(7):1702–13. doi: 10.1038/s41375-025-02635-1 (PMC12208901; doi:10.1038/s41375-025-02635-1)
Supplement: Supplementary file 1 — Supplemental Material [file 41375_2025_2635_MOESM1_ESM.pdf]

Supplementary Methods

Data collection and methods for clinical cohorts

Research participants had either clinical germline cancer risk testing or research-based germline testing for *CHEK2* variants. Testing on probands was via cultured skin fibroblast DNA, and cascade testing of family members used saliva or peripheral blood (PB). Clinical sequencing was performed by the Genetic Services Laboratory (UC) using an augmented whole exome panel or Sanger sequencing. Karyotyping and tumor-based next-generation sequencing (NGS) were performed in a clinical laboratory. Referral and inclusion criteria for the University of Chicago (UC) Hereditary Hematologic Malignancy cohort included: A personal and family history of hematopoietic malignancy, a personal history of hematopoietic malignancy with early onset solid organ malignancy (<50 years) in several family members, personal history of ≥2 cancers including 1 hematopoietic malignancy, an identified possible germline variant on a somatic NGS panel, or an age at diagnosis of hematopoietic malignancy younger than average (e.g., MDS ≤ 40 years). Family members from an identified proband were offered cascade testing and included in the cohort. Referral and inclusion criteria for the UC Hereditary Hematologic Malignancy cohort were in line with the European LeukemiaNet (ELN) 2022 guidelines for clinical germline testing in patients with hematopoietic malignancies. Referrals were both internal from the UC system and external from other institutions in the United States of America. The inclusion and referral criteria for the University of Utah cohort were a personal and family history of solid organ malignancy or identification of a germline variant known to predispose to solid organ malignancies. All patients with pathogenic or likely pathogenic *CHEK2* variants were in the heterozygous state.

Identification of CHEK2 associated HM risk in the UK Biobank

Deleterious variants (Pathogenic and Likely Pathogenic) from cancer risk genes (n=42) were obtained from Clinvar (https://www.ncbi.nlm.nih.gov/clinvar/) which met the following criteria: Germline, Likely pathogenic, Pathogenic, Frameshift, Missense, Nonsense, Splice site, Multiple submitters, Expert panel. UKBiobank participants with the deleterious variants were filtered using Cohort Browser feature of Research Analysis Platform (RAP) by DNA Nexus and participant IDs were downloaded. Following fields were obtained for all the participants(n=502410) in UKBiobank for the analysis: 31-Sex, 34-Year of Birth, 21022 – Age at recruitment, 20160 – Ever Smoked, 40006 - Type of cancer, 40008 – Age of cancer diagnosis, 40009 – Reported Occurrence of Cancer, 40011 – Histology of Cancer Tumor, 40012 - Behaviour of cancer tumor.

Participants with deleterious variants in all other genes were removed for analysis to see the association of participants with deleterious variants CHEK2. Frequency of malignant cancer in Behavior of Cancer Tumor using the International Classification of Diseases for Oncology, 3rd edition (ICD-O) using the following codes {2.0,3.0,6.0,9.0}. (https://biobank.ndph.ox.ac.uk/ukb/ukb/docs/ICDcancermorph.pdf) Distribution of age of onset of first malignant cancer between CHEK2 (P/LP) and CHEK2(WT) was tested using non-parametric Mann-Whitney U-Test. Logistic regression models were applied to assess the association between CHEK2_Status (Pathogenic/Likely Pathogenic = 1, Wildtype = 0) and Malignant Cancer/Heme_Malignant/Cancer/Solid Tumor/Lymphoid Malignancy/Myeloid Malignancy (outcome variable), adjusting for covariates: Age

(continuous), Sex (Male = 1, Female = 0), and Smoking Status (Smoked = 1, Never Smoked = 0).

The penetrance was calculated using the following formula for each age thresholds:

$$\text{Penetrance} = \left(\frac{\text{Number of individuals with the genetic variant and cancer}}{\text{Total number of individuals with the genetic variant}} \right) \times 100$$

42 gene list: *CHEK2, BRCA2, ATM, FANCA, BLM, MPL, FANCD2, FANCC, DDX41, PMS2, NBN, CSF3R, BRCA1, FANCI, MSH6, FANCG, RTEL1, FANCF, FANCM, MLH1, FANCE, NF1, FANCL, MSH2, CDKN2A, TP53, APC, PTPN11, PTEN, CBL, DKC1, SAMD9, ETV6, ANKRD26, CEBPA, FANCB, IKZF1, PAX5, RUNX1, SAMD9L, SRP72, TERC.*

Identification of CHEK2 variants in public AML datasets

Raw RNA-seq reads were aligned to hg38 with STAR aligner and processed with Genome analysis tool kit (GATK)-SplitNCigarReads. Variants were called by ensemble consensus method with (GATK)-Haplotype, FreeBayes, and VarScan 2, and passed if called by ≥2 variant callers without filtering, with annotation by SnpEff. Filtering was then performed for: variant allele frequency (VAF) >0.3, SnpEff MODERATE or HIGH, CADD >10.5, REVEL >0.250 followed by manual curation (Supplemental Table 1). Fusions were annotated from the reported karyotype in the metadata. Recurrent fusions were defined per the European LeukemiaNet (ELN) 2022 as KMT2A rearranged/t(v;11q23.3), MLLT3-KMT2A, PML-RARA, RUNX1-RUNX1T1, CBFB-MYH11, GATA2-MECOM, and DEK2-NUP214.

Generation and characterization of a CHEK2 mouse model

The CHEK2 p.I161T mouse was generated from a simple knock-in allele with T8677C/p.I161T and homology arms targeting exons 3-5 followed injected into murine embryonic stem (ES) cells from C57BL/6 mice in the UC Transgenics and ES facility. Integration was confirmed by Southern blotting and long-range PCR, with subsequent genotyping by Sanger sequencing. Complete blood counts (CBC) were analyzed on a Hemavet 950 (Drew Scientific). Mice were maintained until 24 months or a humane endpoint, determined by regular health checks. Chek2 expression was analyzed in (BM) or spleen by qPCR or Western blot.

To examine the HSPC compartment in *Chek2* p.I161T mice, 2-6 month-old mice had bone marrow extracted by crushing, followed by lineage (Lin) depletion ($\leq 2x10^9$ cells) with the Miltenyi Biotec mouse direct lineage depletion kit and LS columns per manufacturer protocol. Lin^- were stained for Lin, Sca1, cKit, and CD34 and analyzed on a BD LSR Fortessa with bead based FMO compensation on FlowJo software.

For bulk RNA-seq, bone marrow from 5-6 month-old mice were processed and lineage depleted as above, followed by sorting ~100,000-300,000 live Lin^-CD34^+ cells with a BD FACS Aria III directly into RLT+ buffer with β-ME with column-based RNA extraction per manufacturer protocol. Oligo-dT, polyA-depleted mRNA libraries were generated and sequenced on an Illumina NovaSeq PE100 S1 flowcell with ~60M clusters/sample and ~120M paired-end reads per sample. Fastq files were assessed with FastQC (v.0.11.9) followed by transcript quantitation by Salmon (v.1.9.0) with the mm10 reference transcript set, with log2 normalization of counts per million (cpm). Clustering was performed with pheatmap (v.1.0.12) with ward.D2 and correlation clustering method

and distance. Differential gene expression (DGE) and volcano plots generated with DEseq2 (v.1.32.0). DGE were pre-ranked by log2FoldChange and gene set enrichment analysis (GSEA, v.4.2.2) performed with the MSigDB m2.all v.2023 gene set.

Clonal hematopoiesis was assessed by somatic variant identification, performed on DNA derived from PB collected from 8-9 month-old mice. These mice were not serially bled, unlike the mice from the full cohort. Whole exome sequencing (WES) with target coverage of 150X was performed on the Illumina NovaSeq X plus with a S4 flowcell and 100bp paired end reads at the Yale Center for Genome Analysis. Quality was assessed with FastQC and pre-processing per GATK best practices workflow. Variants were called with Mutect2 (GATK v.4.1.3.0) in tumor only mode with GRCm38 reference followed by FilterMutect2Calls and annotation with Annovar (RefGene set, version 2019Oct24). Filtering for somatic variants included removing: Repeat regions, non-exonic, VAF <2% and >40%, present in ≥2 samples, total read depth <20, read depth <3 in alternate allele.

Mouse necropsies were performed on all deceased mice, including gross examination, CBC, blood smears, tissue sections, immunohistochemistry (IHC), and multicolor flow cytometry. PB was stained with Wright-Giemsa, tissue stained with hematoxylin and eosin (H&E). IHC for CD3 and PAX5 was performed on formalin fixed tissue in the UC Human Tissue Research Center. Flow cytometry was performed on fresh or viably frozen PB, BM, spleen, or identified masses with a multicolor panel including CD3, CD4, CD8, CD19, CD11b, CD34, CD117, ±CD45 on a BD LSR Fortessa with bead based FMO compensation analyzed with FlowJo software. A diagnostic label was reached after integrated review of the gross, morphologic, IHC, and flow cytometry findings by two separate investigators. For one mouse that developed an aggressive

$CD3^+CD4^+$ leukemia, PB was transplanted into a sublethally (450 rads) irradiated CD45.1 recipient mouse.

For B/T clonotype sequencing, column-based RNA extraction was performed on viably frozen PB, BM, spleen, or malignant mass from endpoint mice and subjected to library preparation as per the NEBNext Mouse Immune Sequencing Kit manufacturer protocol. Libraries had Bioanalyzer QC performed and sequencing on an Illumina MiSeq with 300bp paired end reads and a 600 cycle V3 MiSeq reagent kit with an index read between the first and second read. Fastqs were assessed with FastQC and processed by MiXCR (v.4.4.2) with the NEBNext mouse preset parameters to produce B-cell and T-cell clonotype calls. Clonality results were qualitatively plotted and quantitatively expressed as the percentile rank of the individual clone that comprised a ≥50% cumulative fraction of the total clonal burden within a single sample.

Statistics and Institutional Approvals

This study was approved by the UC Institutional Review Board (protocol #11-0014), and all research participants gave written informed consent. Mice were maintained in the UC animal facility (protocol #71370). Comparisons were by unpaired t-test and survival calculated using the Kaplan-Meier method with $P<0.05$ considered significant, except where otherwise specified. Transgenic mice were generated at the University of Chicago Transgenics Facility and sequencing performed at the University of Chicago Genomics core, except for the WES which was performed at the Yale University Sequencing core. Flow cytometry and cell sorting was performed at the University of Chicago flow cytometry core.

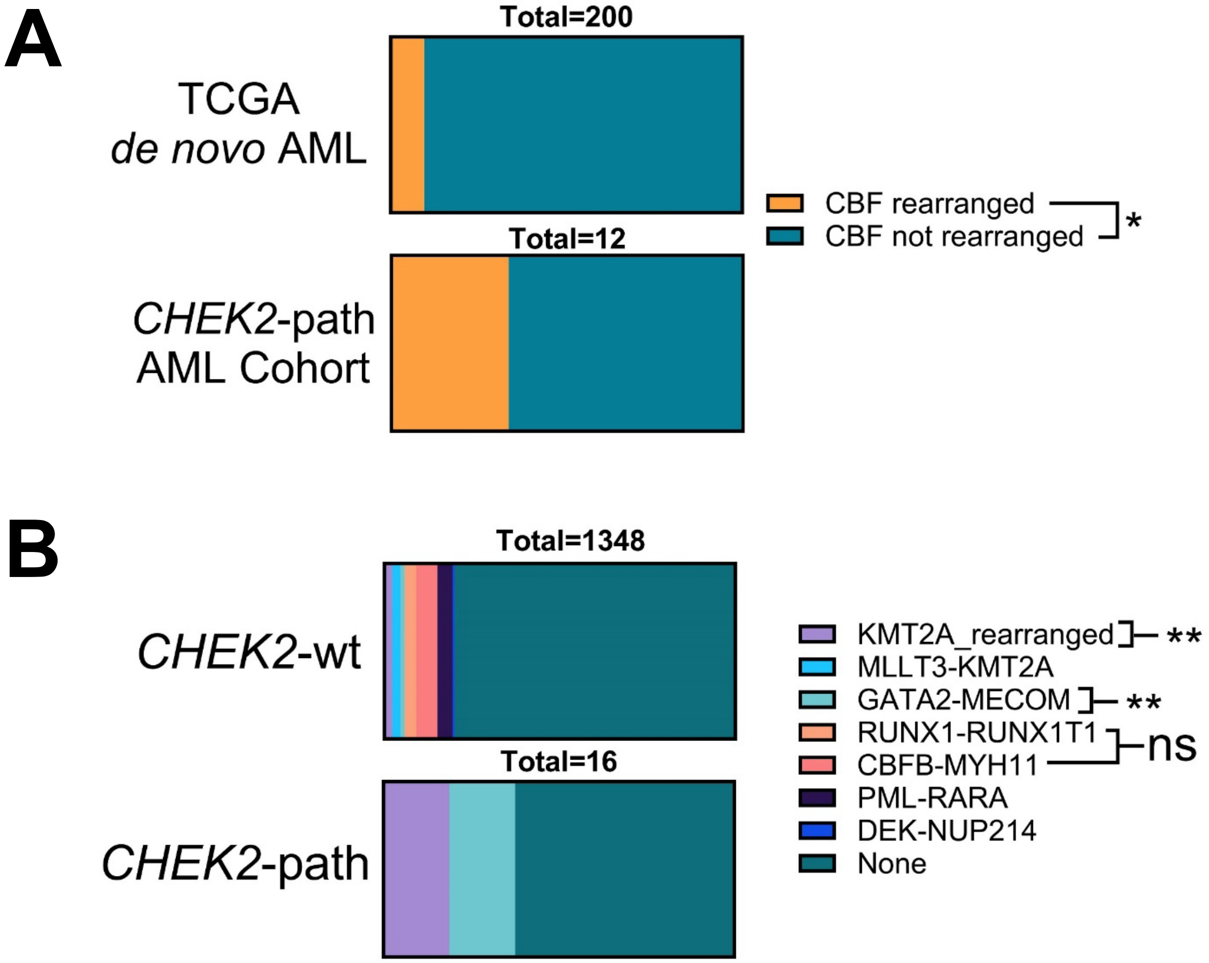

Supplemental Figure 1. Cytogenetic features of patients with *CHEK2* variants. (A) University of Chicago (UC) cohort - comparison between the number of AML patients with core binding factor (CBF) fusions from the TCGA, a *de novo* AML cohort (top) and those with P/LP germline *CHEK2* variants (*CHEK2*-path) (bottom). CBF rearrangements were defined as CBFB-MYH11 or RUNX1-RUNX1T1 (B) Public RNA-seq datasets – Fusions, as identified from the clinical karyotypes reported in the metadata of AML patients who are *CHEK2*-wt vs. *CHEK2*-path.

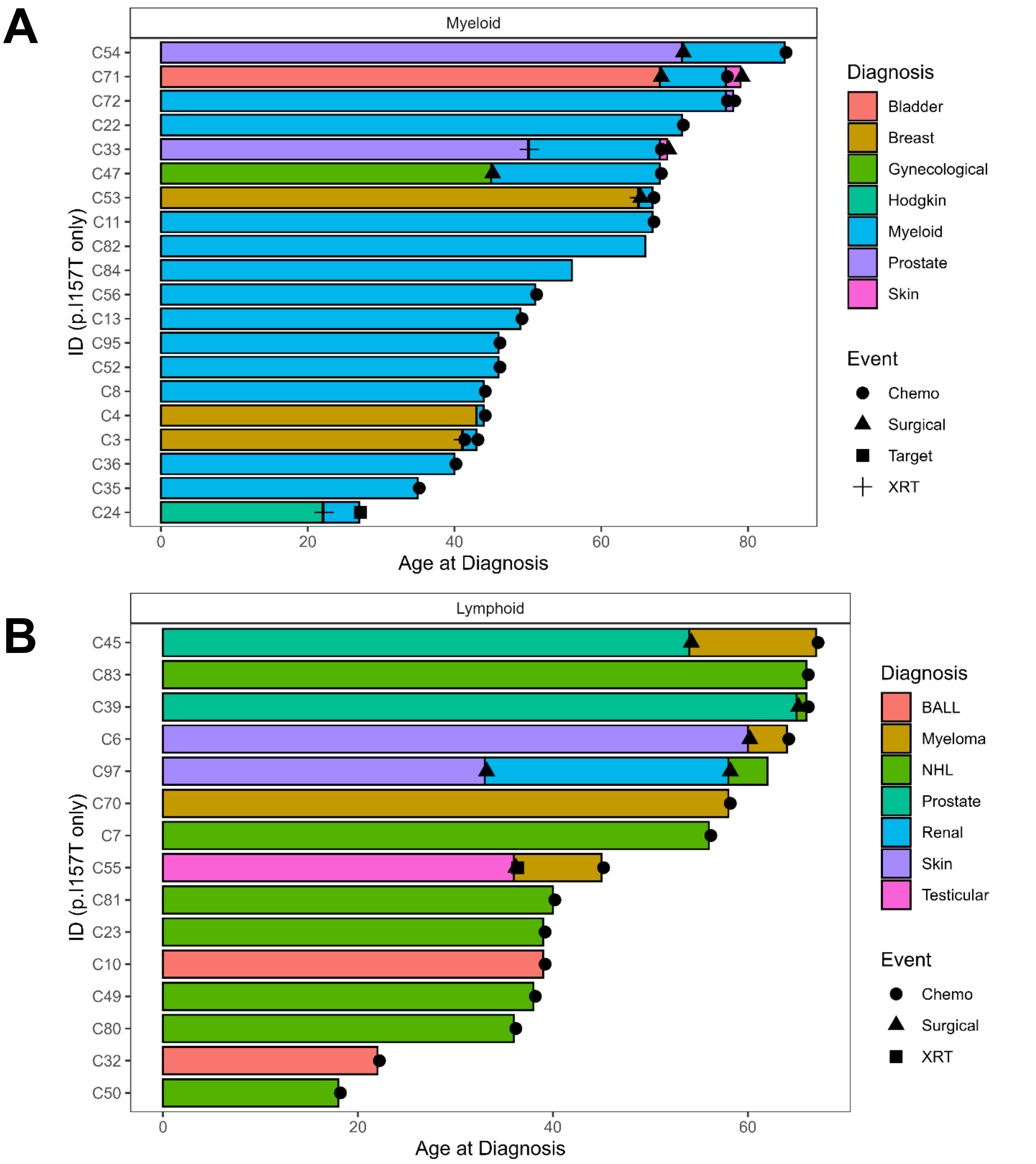

Supplemental Figure 2. Treatment course for *CHEK2* mutated patients with hematopoietic malignancy. Swimmers plots demonstrate the order of malignancies and treatments received in those with the germline *CHEK2* p.I157T allele who developed (A) myeloid or (B) lymphoid malignancies.

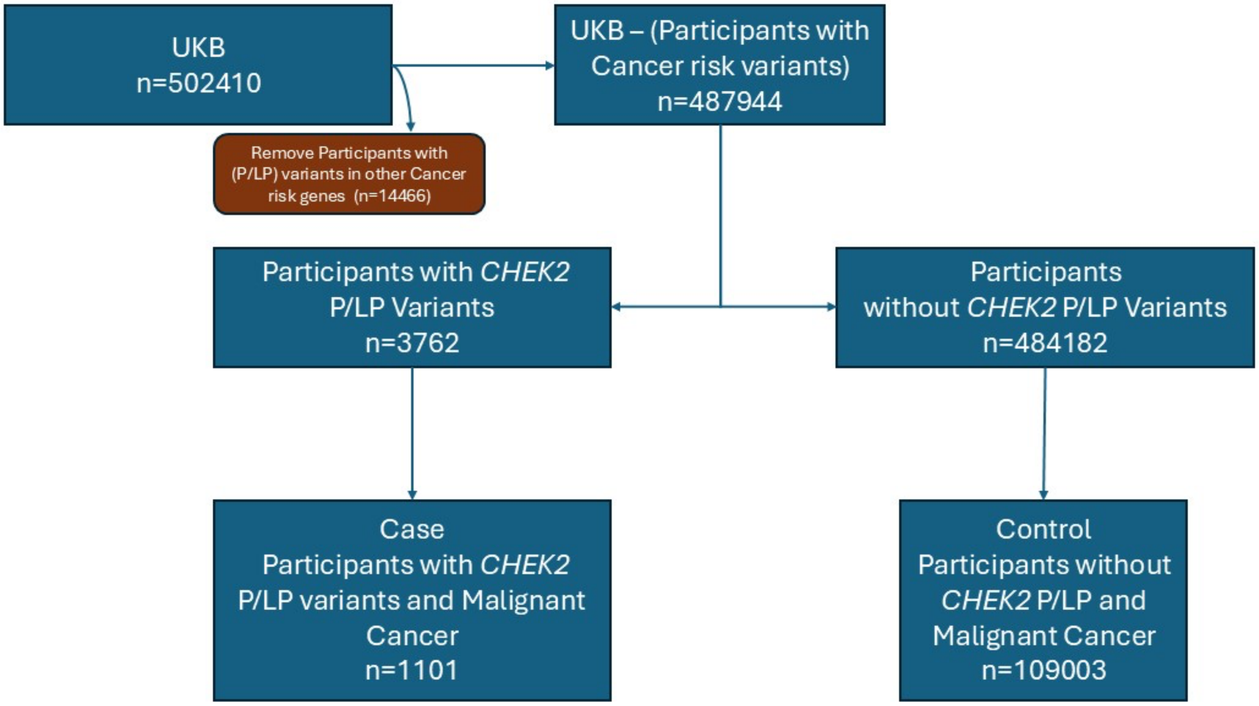

Supplemental Figure 3. Flow diagram for disposition of UK Biobank participants. All UK Biobank (UKBB) participants were assessed and patients with a known pathogenic (P) or likely pathogenic (LP) variant in a non-*CHEK2* cancer predisposition genes were removed. This cohort was then divided into patients with a *CHEK2* P/LP variant (defined as a P/LP deposit in ClinVar) and those without, and those two groups subdivided into participants with a malignant cancer and without a malignant cancer.

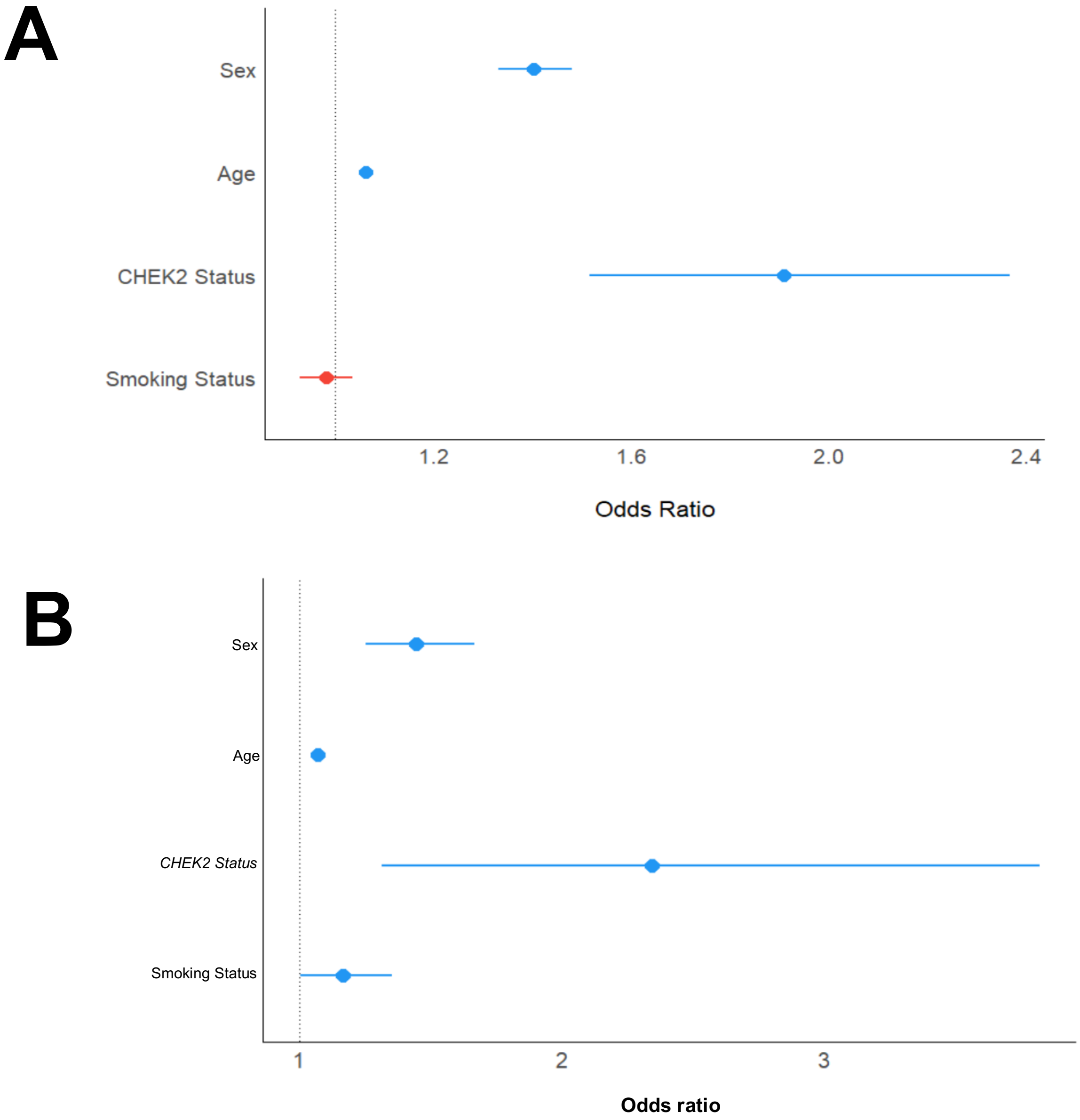

Supplemental Figure 4. Multivariable logistic regression for UK Biobank participants for hematopoietic malignancy and myeloid malignancy. (A) Multivariable logistic regression for *CHEK2* status and hematologic malignancy (HM) in UKBB participants (*CHEK2* status: OR 1.91, 95% CI 1.52-2.37, *P*<0.001). (B) Multivariable logistic regression for *CHEK2* status and myeloid malignancy in UKBB participants (*CHEK2* status: OR 2.34, 95% CI 1.31-3.82, *P*=0.002).

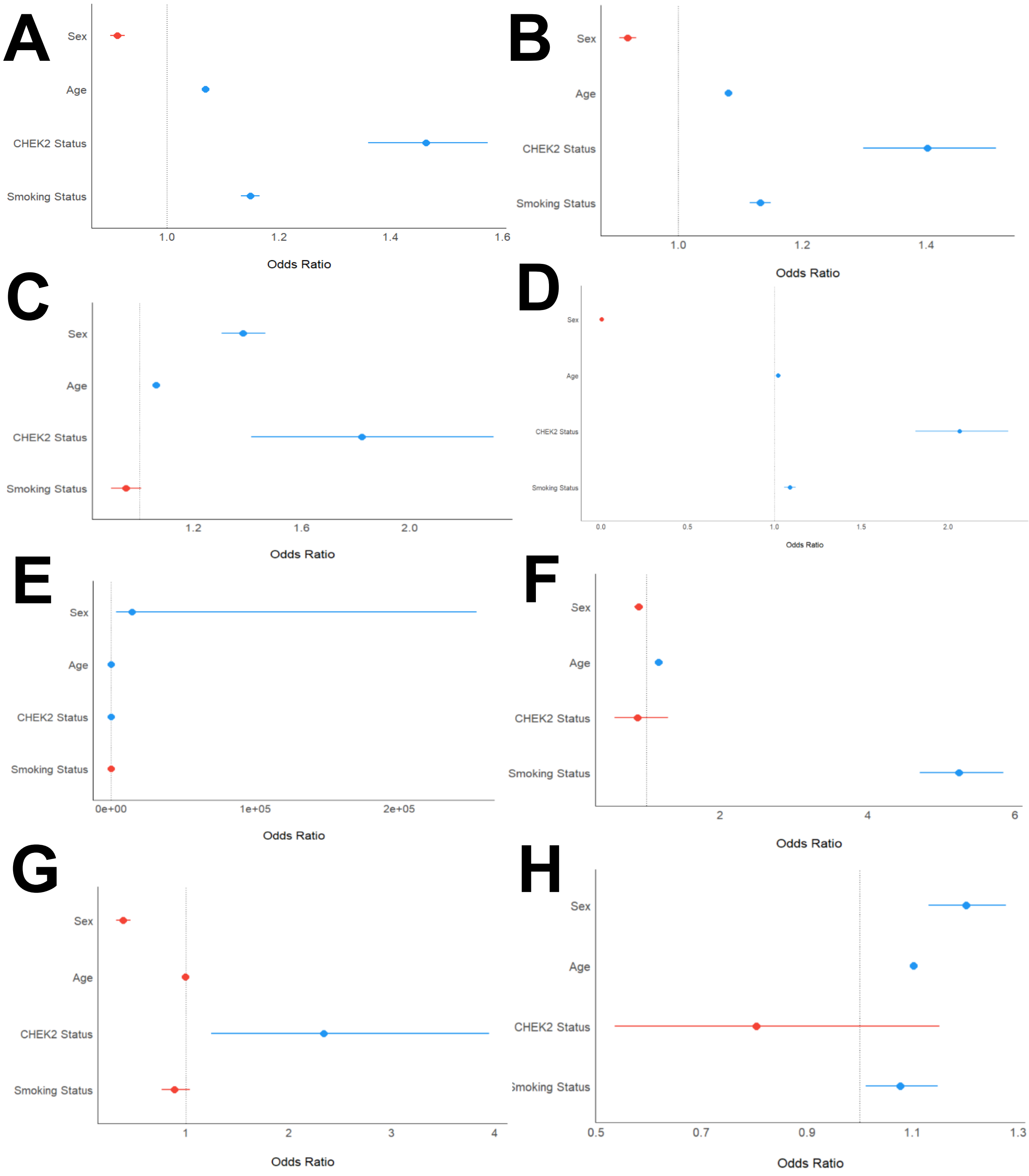

Supplemental Figure 5. Multivariable logistic regression for UK Biobank participants for other malignancies. Logistic regression was performed for UK Biobank participants including the variables of Sex, Age, *CHEK2* status, and smoking status. (A) Any malignant cancer. (B) Solid tumor. (C) Lymphoid malignancy. (D) Breast neoplasm. (E) Prostate neoplasm. (F) Lung neoplasm. (G) Thyroid neoplasm. (H) Colon neoplasm. Odds ratio, 95% confidence intervals, and *P*-values are demonstrated in Supplemental Table 10.

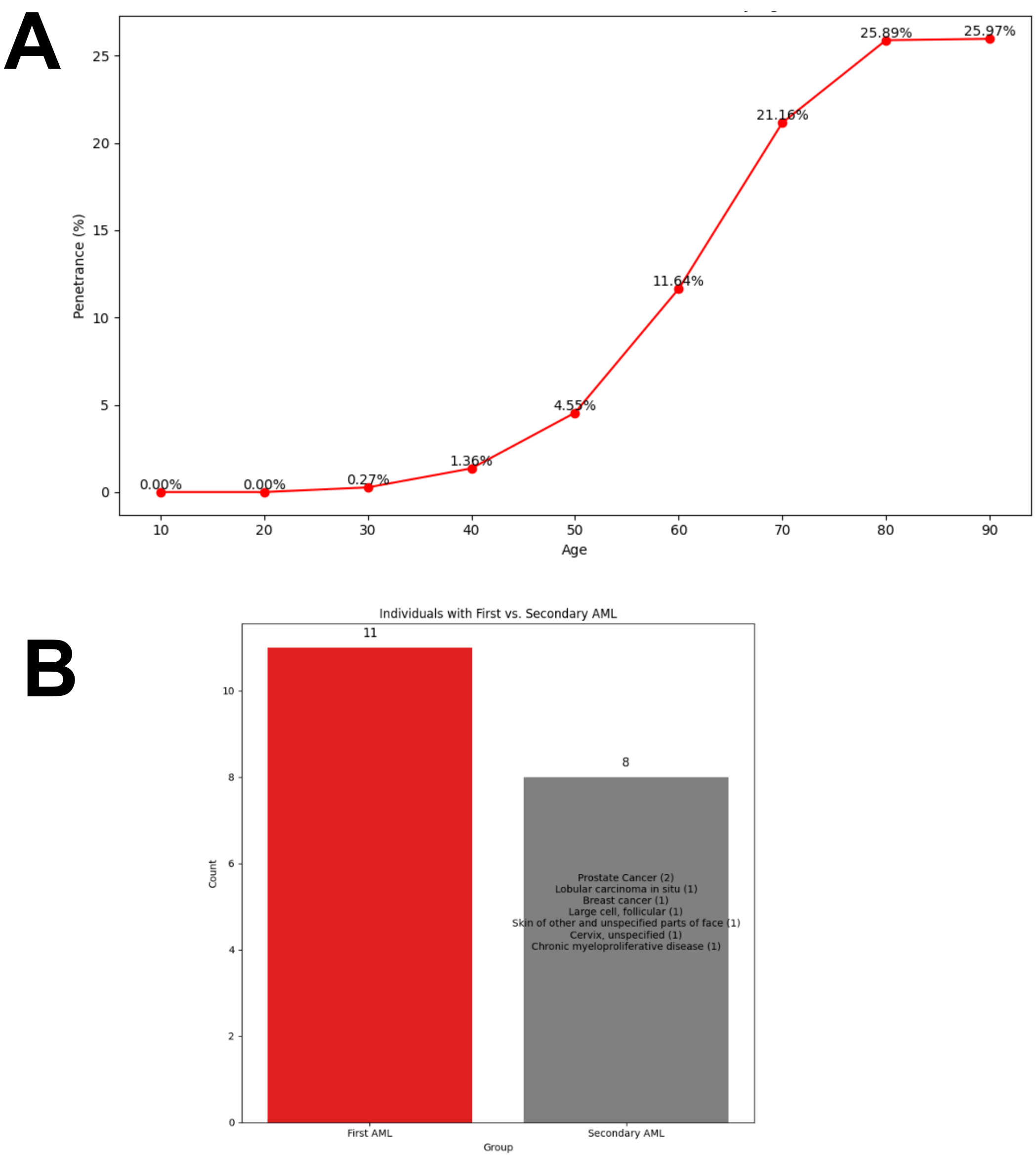

Supplemental Figure 6. UK Biobank (UKBB) prevalence of solid tumors and AML. (A) Prevalence of solid tumor in UKBB participants by age. (B) Amongst 19 patients with *CHEK2* pathogenic/likely pathogenic variants (*CHEK2*-path), 58% (11/19) had de novo AML and 42% (8/19) had secondary AML.

A

Sequences:
Sequence 1: CHK2_HUMAN (O96017) (543 residues)
Sequence 2: CHK2_MOUSE (Q9Z265) (546 residues)

Parameters:
Comparison matrix: BLOSUM62
Number of alignments computed: 20
Gap open penalty: 12
Gap extension penalty: 4

```
83.5% identity in 541 residues overlap; Score: 2285.0; Gap frequency: 2.2%

O96017|CHK     9 AQQSHGSSACSQPHGSVTQSQGSSSQS------QGISSSSTSTMPNSSQSSHSSSGTLSS
Q9Z265|CHK    12 SSKAHDSASCSQSQGGFSQPQGTPSQLHELSQYQGSSSSSTGTVPSSSQSSHSSSGTLSS
                     * *  ***  *   * **  **       ** ***** * * **************

O96017|CHK    63 LETVSTQELYSIPEDQEPEDQEPEEPTPAPWARLWALQDGFANLECVNDNYWFGRDKSCE
Q9Z265|CHK    72 LETVSTQELCSIPED-----QEPEEPGPAPWARLWALQDGFSNLDCVNDNYWFGRDKSCE
                 ********* *****     ****** ************** ** ***************

O96017|CHK   123 YCFDEPLLKRTDKYRTYSKKHFRIFREVGPKNSYIAYIEDHSGNGTFVNTELVGKGKRRP
Q9Z265|CHK   127 YCFDGPLLRRTDKYRTYSKKHFRIFREMGPKNCYIVYIEDHSGNGTFVNTELIGKGKRCP
                 **** *** ****************** **** ** **************** ***** *

O96017|CHK   183 LNNNSEIALSLSRNKVFVFFDLTVDDQSVYPKALRDEYIMSKTLGSGACGEVKLAFERKT
Q9Z265|CHK   187 LSNNSEIALSLCRNKVFVFFDLTVDDQSVYPKELRDEYIMSKTLGSGACGEVKMAFERKT
                 * ********* ******************* ********************* ******

O96017|CHK   243 CKKVAIKIISKRKFAIGSAREADPALNVETEIEILKKLNHPCIIKIKNFFDAEDYYIVLE
Q9Z265|CHK   247 CQKVAIKIISKRRFALGSSREADTAPSVETEIEILKKLNHPCIIKIKDVFDAEDYYIVLE
                 * ********** ** ** **** *  ********************  ***********

O96017|CHK   303 LMEGGELFDKVVGNKRLKEATCKLYFYQMLLAVQYLHENGIIHRDLKPENVLLSSQEEDC
Q9Z265|CHK   307 LMEGGELFDRVVGNKRLKEATCKLYFYQMLVAVQYLHENGIIHRDLKPENVLLSSQEEDC
                 ********* ******************** *****************************

O96017|CHK   363 LIKITDFGHSKILGETSLMRTLCGTPTYLAPEVLVSVGTAGYNRAVDCWSLGVILFICLS
Q9Z265|CHK   367 LIKITDFGQSKILGETSLMRTLCGTPTYLAPEVLVSNGTAGYSRAVDCWSLGVILFICLS
                 ******** *************************** ***** *****************

O96017|CHK   423 GYPPFSEHRTQVSLKDQITSGKYNFIPEVWAEVSEKALDLVKKLLVVDPKARFTTEEALR
Q9Z265|CHK   427 GYPPFSEHKTQVSLKDQITSGKYNFIPEVWTDVSEEALDLVKKLLVVDPKARLTTEEALN
                 ******** *********************  *** **************** ******

O96017|CHK   483 HPWLQDEDMKRKFQDLLSEENESTALPQVLAQPSTSRKRPREGEAEGAETTKRPAVCAAV
Q9Z265|CHK   487 HPWLQDEYMKKKFQDLLVQEKNSVTLPVAPAQTS-SQKRPLELEVEGMPSTKRLSVCGAV
                 ******* ** ******  *  *  **   ** * * *** * * **   ***  ** **

O96017|CHK   543 L
Q9Z265|CHK   546 L
                 *
```

B

```
                                CHEK2 c.470T>C
Homo sapiens 5'-TGGGTCCTAAAAACTCTTACAT>CTGCATACATAGAAGATCACAGTGGCAATGGAACCTTTGTAAAT-3'
                |||| |||||||| | ||||||   | ||||||||| ||||||||||| ||||||||||| ||||||
Mus musculus 5'-TGGGCCCTAAAAATTGTTACAT>CCGTATACATAGAGGATCACAGTGGAAATGGAACCTTCGTAAAT-3'
                                Chek2 c.521T>C

                                CHK2 p.I157T
Homo sapiens    YRTYSKKHFRIFREVGPKNSYI>TAYIEDHSGNGTFVNTELVGKGKRRPLNNNSEIALSLSRNKVFV
                |||||||||||||| |||| ||   |||||||||||||||| ||||| || ||||||||| ||||||
Mus musculus    YRTYSKKHFRIFREMGPKNCYI>TVYIEDHSGNGTFVNTELIGKGKRCPLSNNSEIALSLCRNKVFV
                                Chk2 p.I161T
```

Supplemental Figure 7. Alignment of human and murine *CHEK2* gene and protein. (A) Human *CHEK2* and mouse *Chek2* have a 83.5% homology (ExPasy). A protein alignment of human CHK2 and mouse CHK2 shows the homology around the human Ile 157 amino acid, equivalent to the Ile 161 amino acid in mouse. (B) The human *CHEK2* c.470T>C change and the equivalent mouse *Chek2* c.521T>C change produces either the human CHK2 p.I157T or mouse Chk2 p.I161T amino acid change.

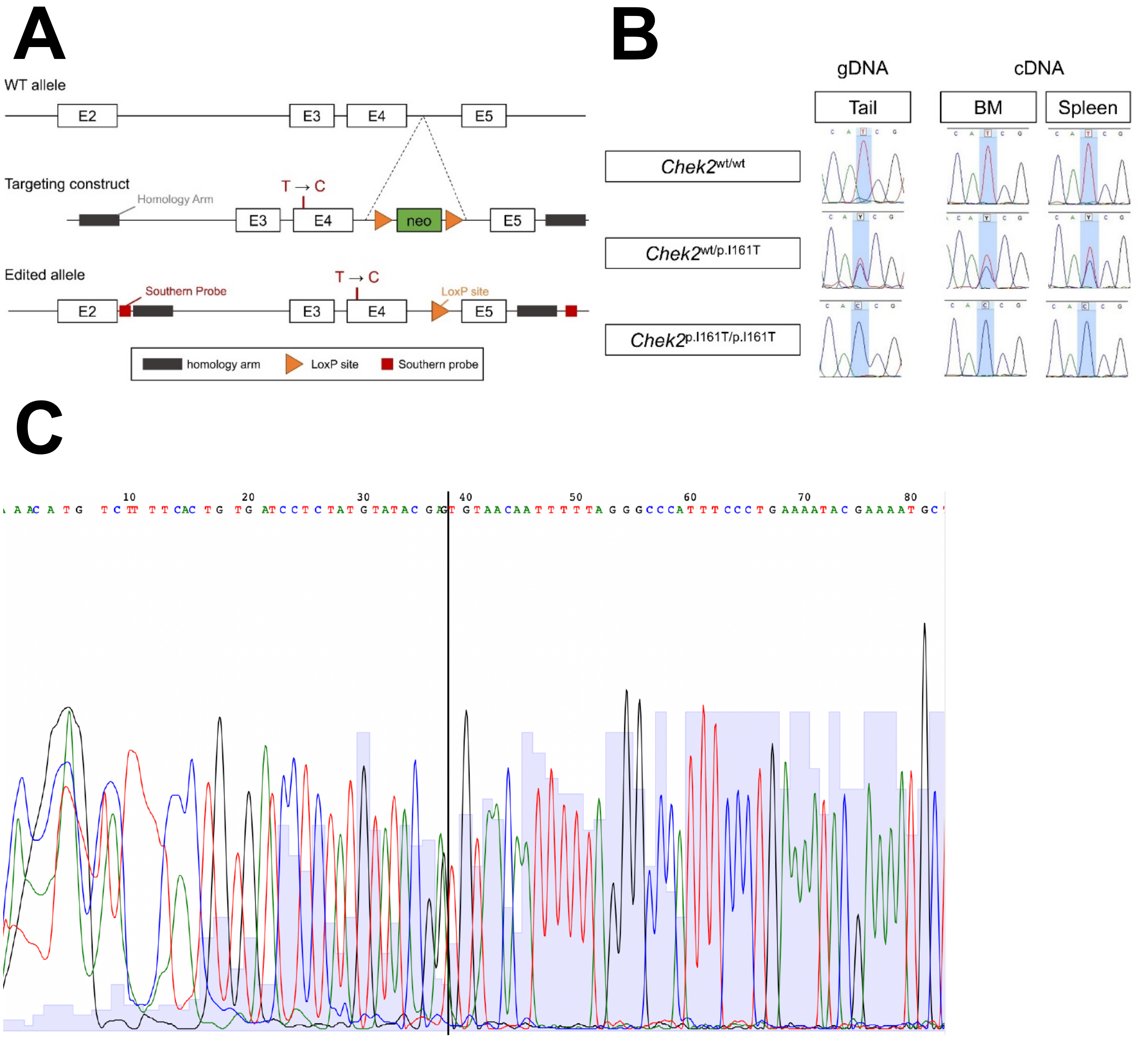

Supplemental Figure 8. Generation of a simple knock-in *Chek2* p.I161T mouse model. (A) Schematic of the targeting construct used to generate the *Chek2* p.I161T allele. The exons (E) are shown with rectangles, the targeting construct with the T→C nucleotide change targeting exon 4 (E4) is shown. A loxP flanked (orange triange) neomycin (neo) cassette allele was included for selection. The final edited allele is shown at the bottom. (B) Sanger sequencing chromatograms are shown from tail genomic DNA (gDNA) as well as bone marrow (BM) and spleen cDNA for wild-type (*Chek2*$^{wt/wt}$), heterozygous (*Chek2*$^{p.I161T/wt}$), and homozygous (*Chek2*$^{p.I161T/p.I161T}$) mice with the base change highlighted. (C) Sanger sequencing chromatograms are shown from *Chek2*$^{p.I161T/wt}$ mouse spleen cDNA generated from extracted RNA demonstrates expression of both the wt and p.I161 alleles.

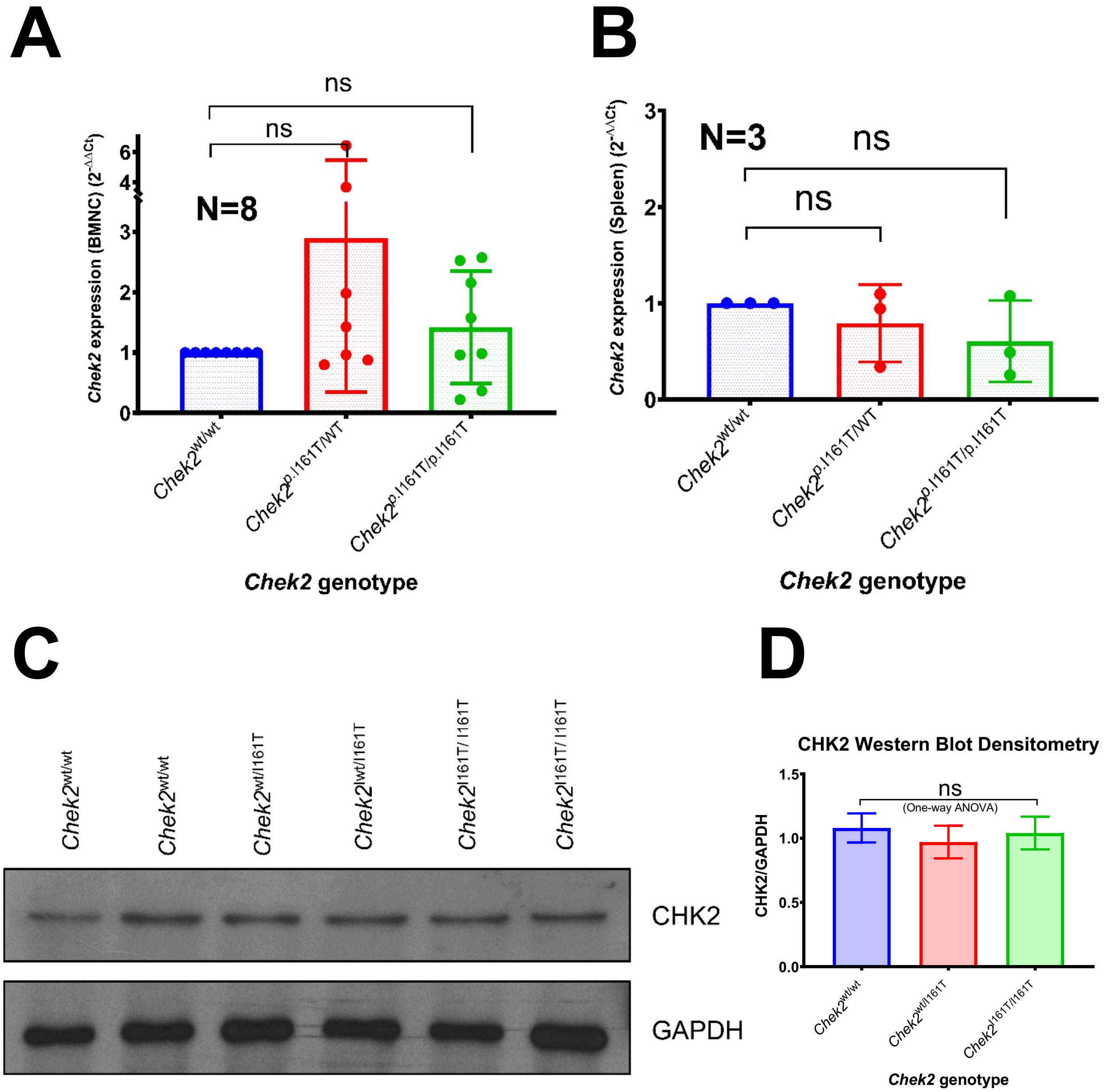

Supplemental Figure 9. Baseline *Chek2* expression in p.I161T mice. (A) There was no statistical difference in *Chek2* expression by quantitative PCR (qPCR) in bone marrow nucleated cells (BMNC) between wild-type (*Chek2*$^{wt/wt}$), heterozygous (*Chek2*$^{p.I161T/wt}$) (P=0.054), and homozygous (*Chek2*$^{p.I161T/p.I161T}$) (P=0.225) mice, although some outliers were seen in the heterozygous group. (B) There was no statistical difference in *Chek2* expression by quantitative PCR (qPCR) in spleen between wild-type (*Chek2*$^{wt/wt}$), heterozygous (*Chek2*$^{p.I161T/wt}$) (P=0.420), and homozygous (*Chek2*$^{p.I161T/p.I161T}$) (P=0.182) mice. (C-D) A Western Blot demonstrates no difference in protein expression between (*Chek2*$^{wt/wt}$), heterozygous (*Chek2*$^{p.I161T/wt}$) , and homozygous (*Chek2*$^{p.I161T/p.I161T}$) mice (P=0.680, ANOVA). (ns, not significant, * $P < 0.05$, ** $P<0.01$, *** $P<0.001$).

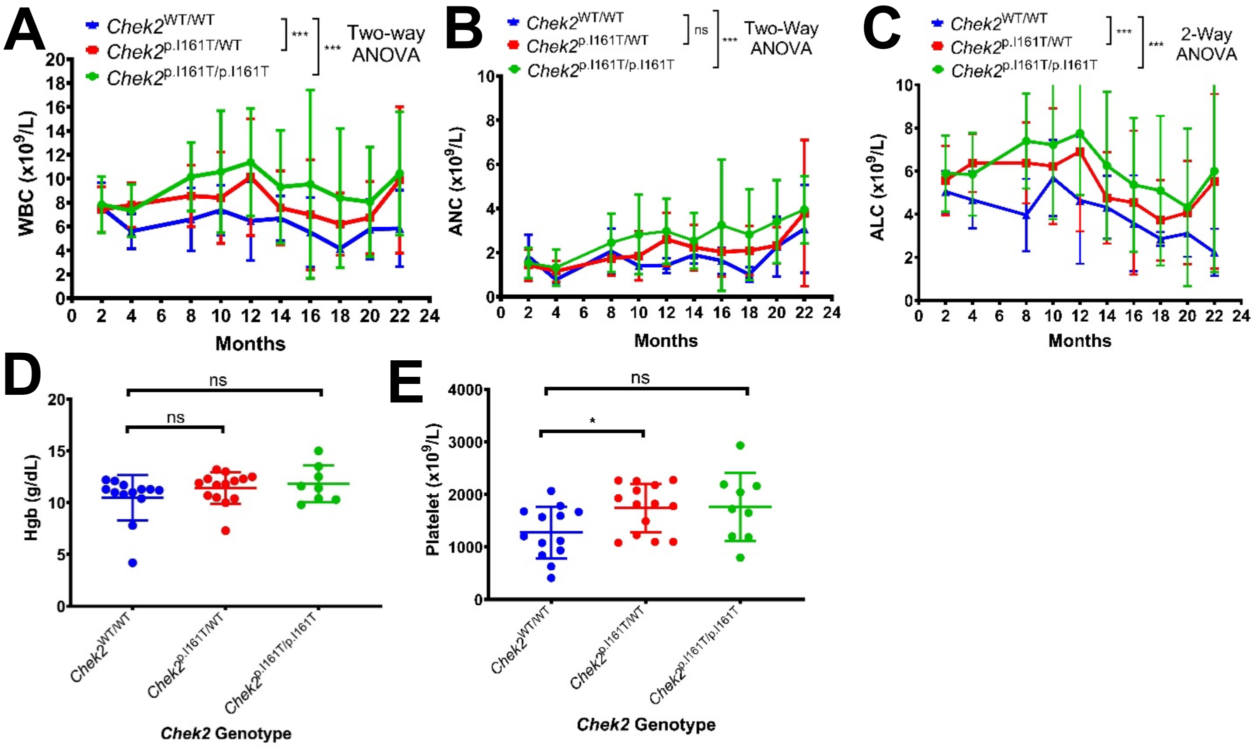

Supplemental Figure 10. Complete blood count (CBC) parameters of a *Chek2* p.I161T mouse model and survival. (A) Total white blood cell counts (WBC) vs. time in *Chek2*$^{wt/wt}$ (blue), *Chek2*$^{p.I161T/wt}$ (red), vs. *Chek2*$^{p.I161T/p.I161T}$(green) mice. (B) Absolute neutrophil count (ANC) vs. time in *Chek2*$^{wt/wt}$ (blue), *Chek2*$^{p.I161T/wt}$ (red), vs. *Chek2*$^{p.I161T/p.I161T}$(green) mice. (C) Absolute lymphocyte count (ALC) vs. time in *Chek2*$^{wt/wt}$ (blue), *Chek2*$^{p.I161T/wt}$ (red), vs. *Chek2*$^{p.I161T/p.I161T}$(green) mice. (D) Hemoglobin (Hgb) levels at endpoint in *Chek2*$^{wt/wt}$ (blue), *Chek2*$^{p.I161T/wt}$ (red), vs. *Chek2*$^{p.I161T/p.I161T}$(green) mice. (E) Platelet levels at endpoint in *Chek2*$^{wt/wt}$ (blue), *Chek2*$^{p.I161T/wt}$ (red), vs. *Chek2*$^{p.I161T/p.I161T}$(green) mice. (ns, not significant, * $P < 0.05$, ** $P<0.01$, *** $P<0.001$).

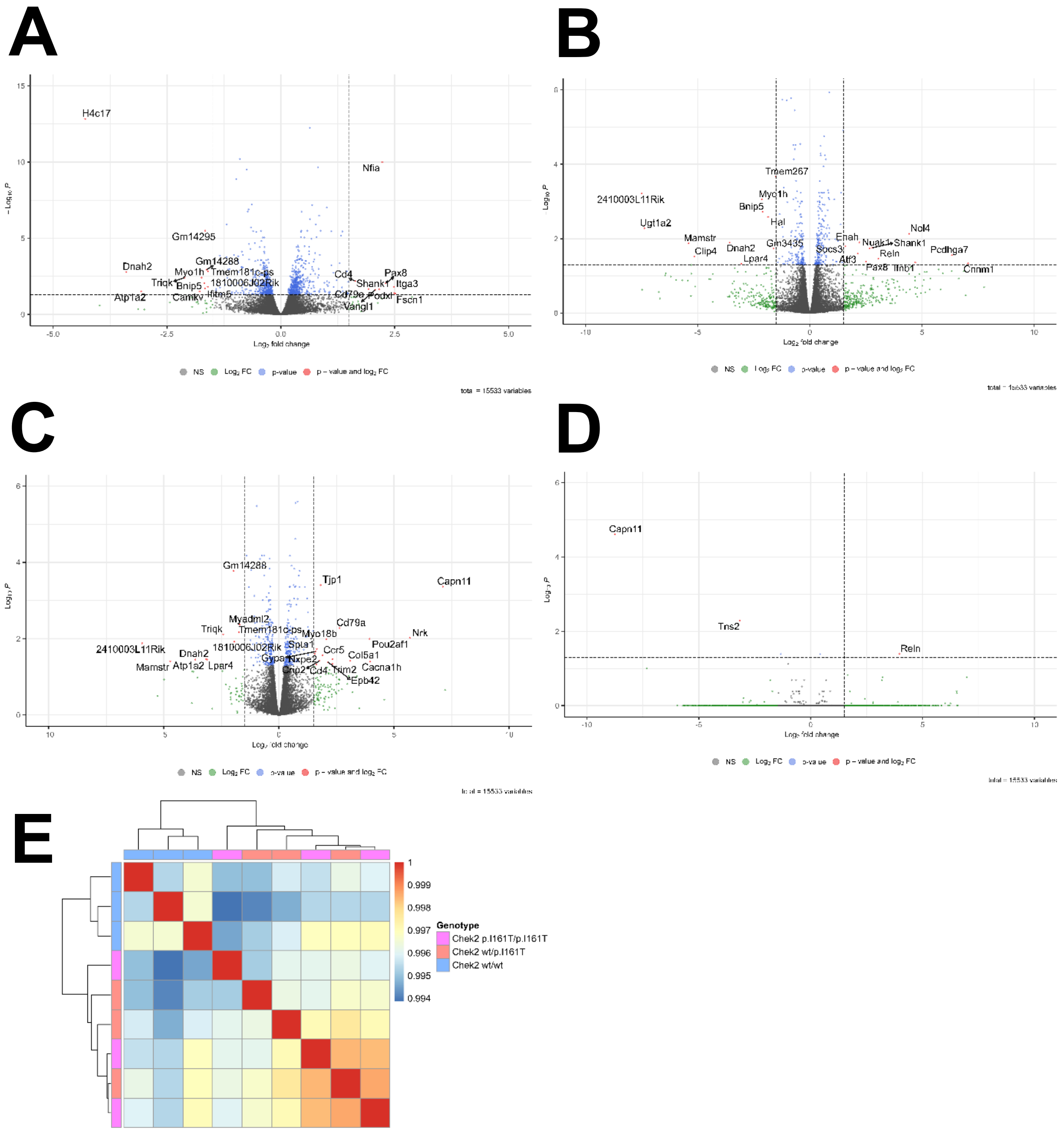

Supplemental Figure 11. Volcano plot showing differential gene expression (DGE) in Lin$^-$CD34$^+$ cells from *Chek2* mice. (A) Wild-type (*Chek2*$^{wt/wt}$) *vs.* mutated (*Chek2*$^{p.I161T/wt}$ or *Chek2*$^{p.I161T/p.I161T}$) mice, (B) Wild-type (*Chek2*$^{wt/wt}$) *vs.* heterozygous (*Chek2*$^{p.I161T/wt}$) mice, (C) Wild-type (*Chek2*$^{wt/wt}$) *vs.* and homozygous (*Chek2*$^{p.I161T/p.I161T}$) mice, (D) Heterozygous (*Chek2*$^{p.I161T/wt}$) *vs.* homozygous (*Chek2*$^{p.I161T/p.I161T}$) mice. There was significant difference between wild-type and heterozygous or homozygous mice, but no DGE between heterozygous and homozygous mice. (E) Heatmap with unsupervised clustering for RNA-seq of Lin-CD34+ cells from young (age 5-6 month) *Chek2* mice.

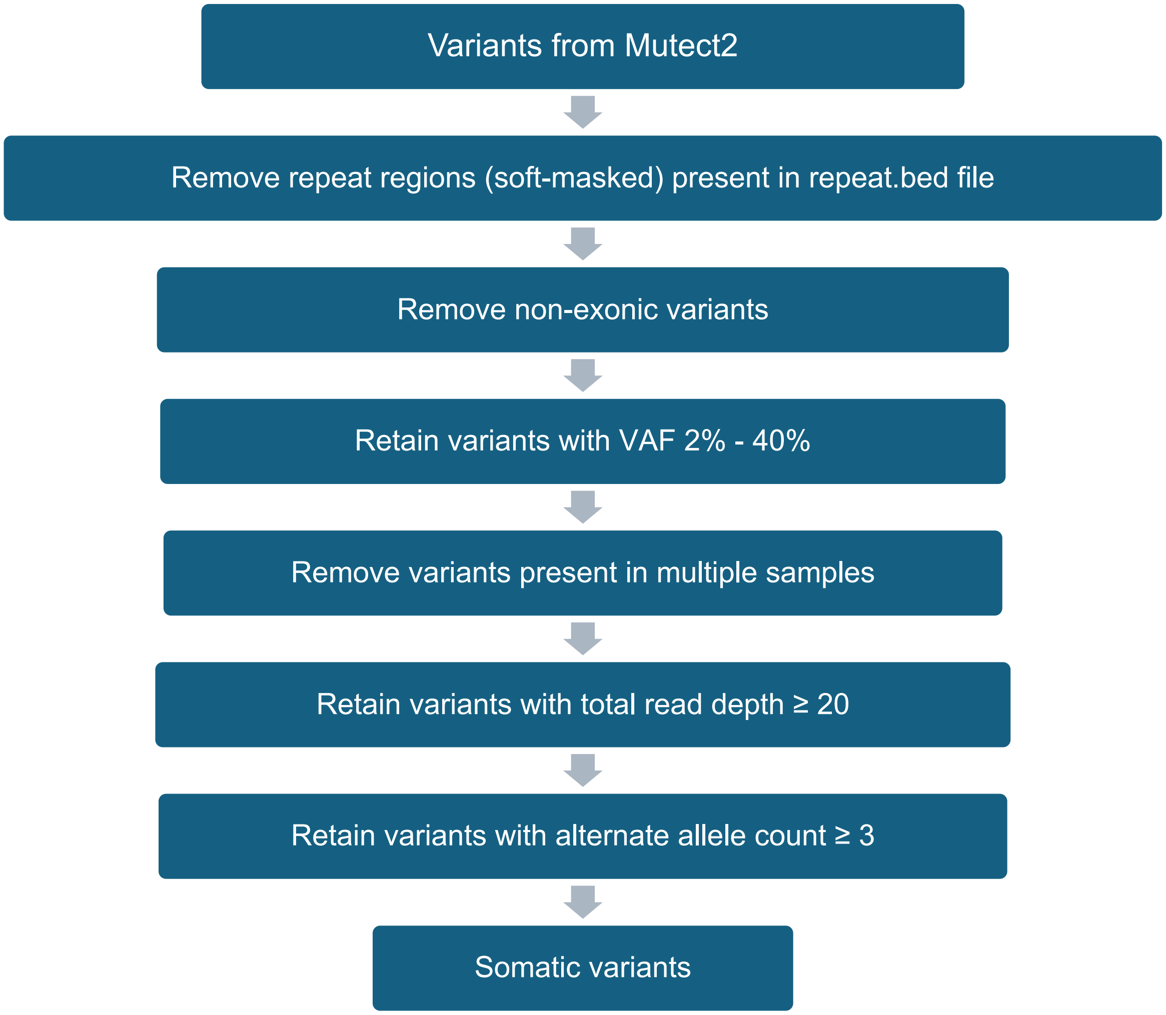

Supplemental Figure 12. Criteria for selecting somatic variants. Workflow for selection of somatic variants from protein coding regions that meet our established parameters for characteristics such as variant allele frequency (VAF), total read depth (DP), and allele count of alternate allele (AC).

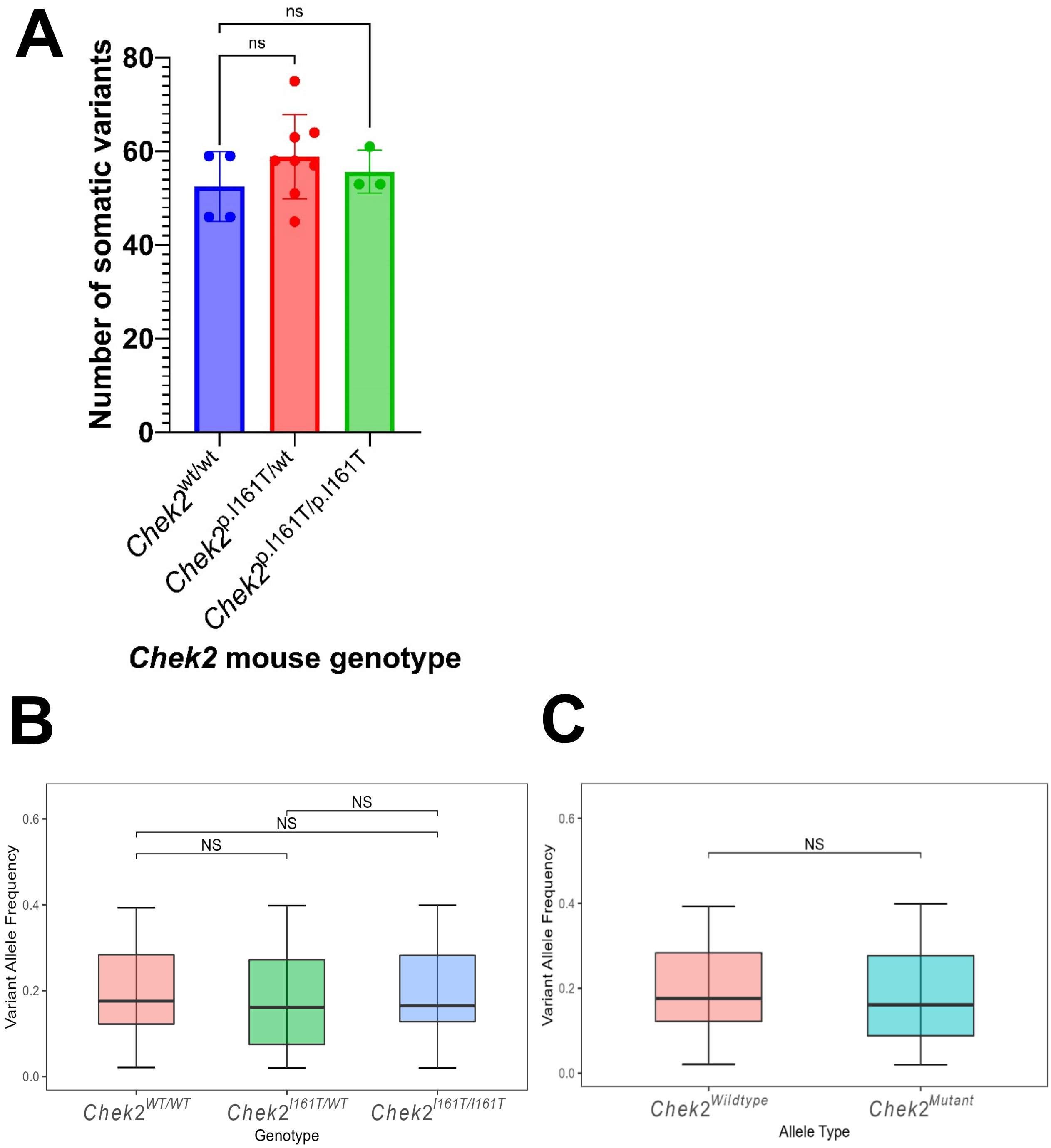

Supplemental Figure 13. Comparison of variant allele frequencies. (A) We did not observe any significant difference in the number of somatic variants between wild-type (*Chek2*$^{wt/wt}$), heterozygous (*Chek2*$^{p.I161T/wt}$), and homozygous (*Chek2*$^{p.I161T/p.I161T}$) mice. (B) Variant allele frequency *vs.* mouse genotype. (C) Variant allele frequency *vs.* allele type. Abbreviation used: NS, not significant. (ns, not significant, * $P < 0.05$, ** $P<0.01$, *** $P<0.001$).

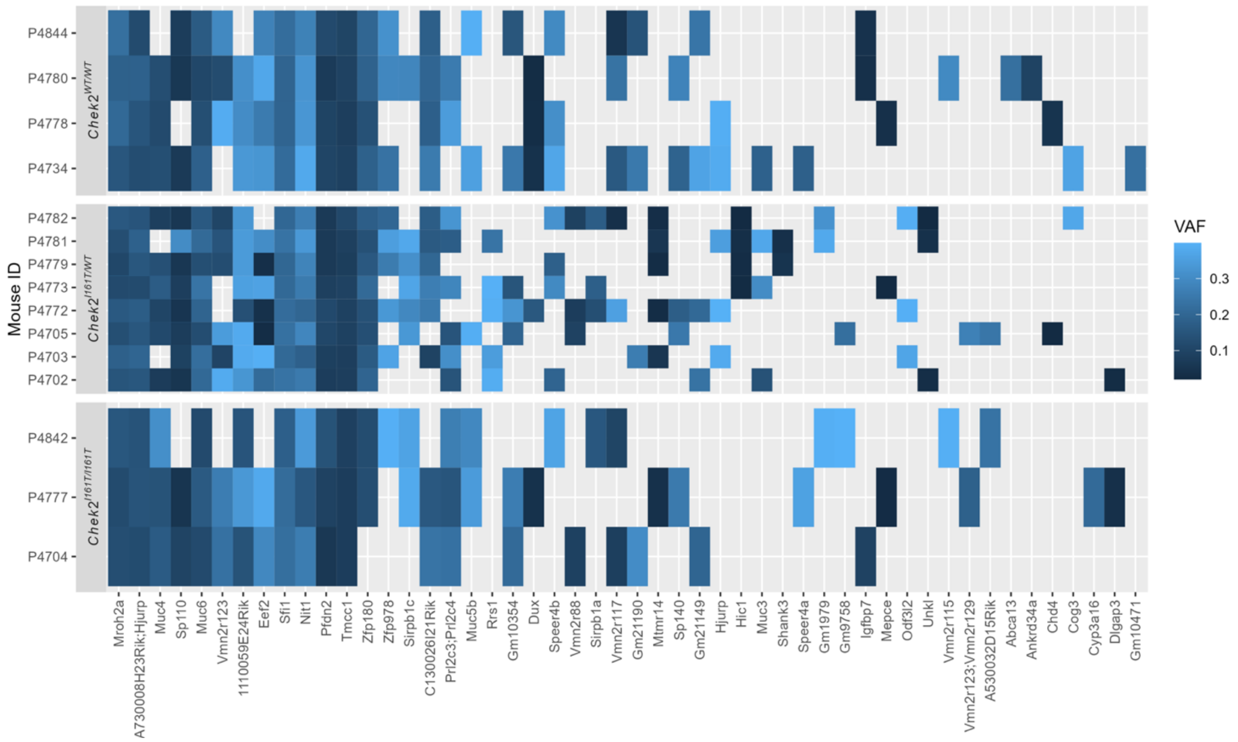

Supplemental Figure 14. Heatmap of the top 100 genes obtained from somatic variant calling. Intensity of the tiles is based on the variant allele frequency (VAF).

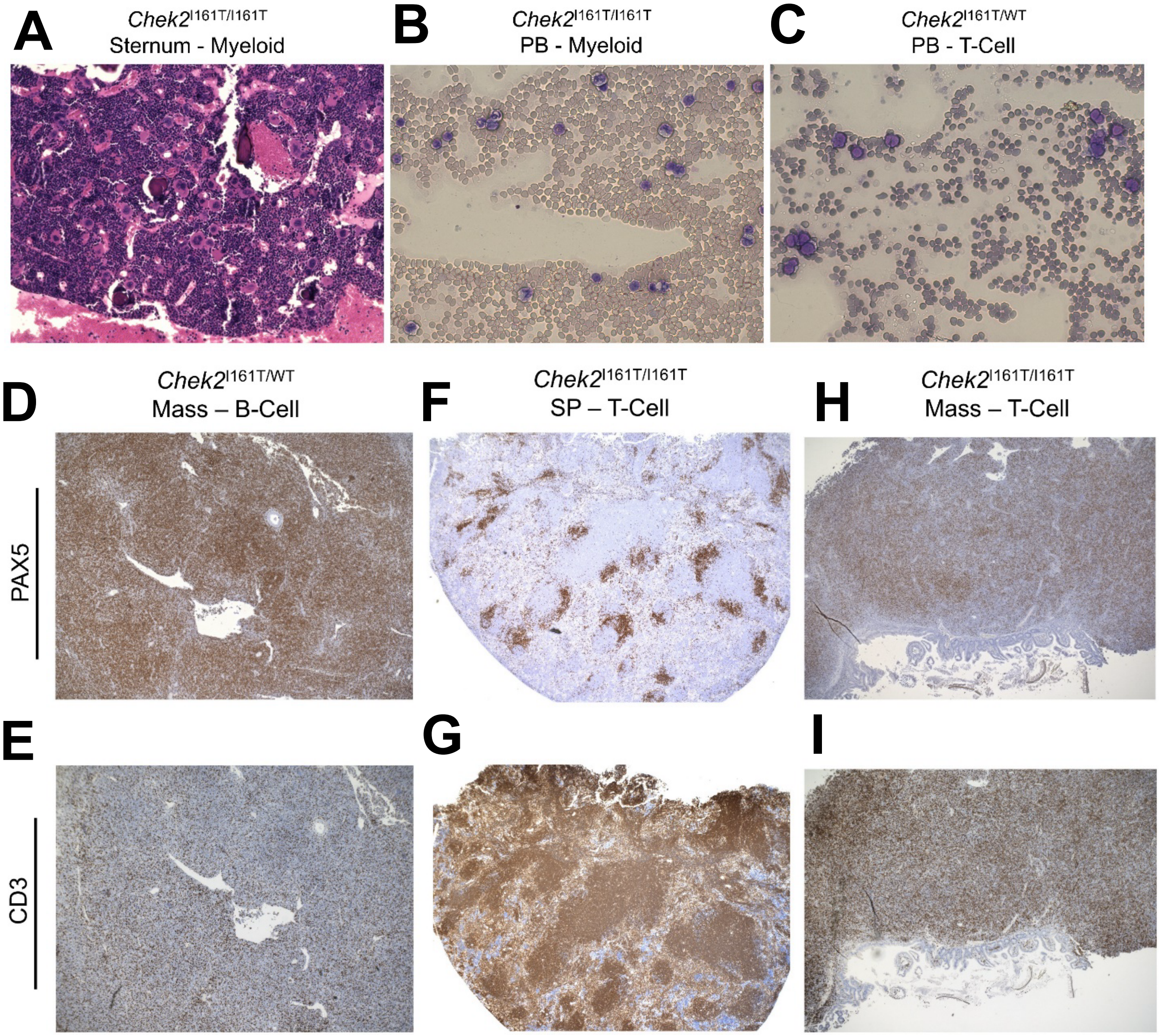

Supplemental Figure 15. Representative histology and immunohistochemistry (IHC) for endpoint *Chek2* mice. Mice were sacrificed at either 24 months or at humane endpoint and subjected to comprehensive necropsy. (A-C) Representative hematoxylin and eosin (H&E) or Giemsa-stained slide images from heterozygous (*Chek2*$^{p.I161T/wt}$) and homozygous (*Chek2*$^{p.I161T/p.I161T}$) mice with features suggestive of myeloid (A, B) or lymphoid (C) malignancy. (D-I) Representative IHC slide images from heterozygous (*Chek2*$^{p.I161T/wt}$) and homozygous (*Chek2*$^{p.I161T/p.I161T}$) mice with features suggestive of lymphoid malignancy.

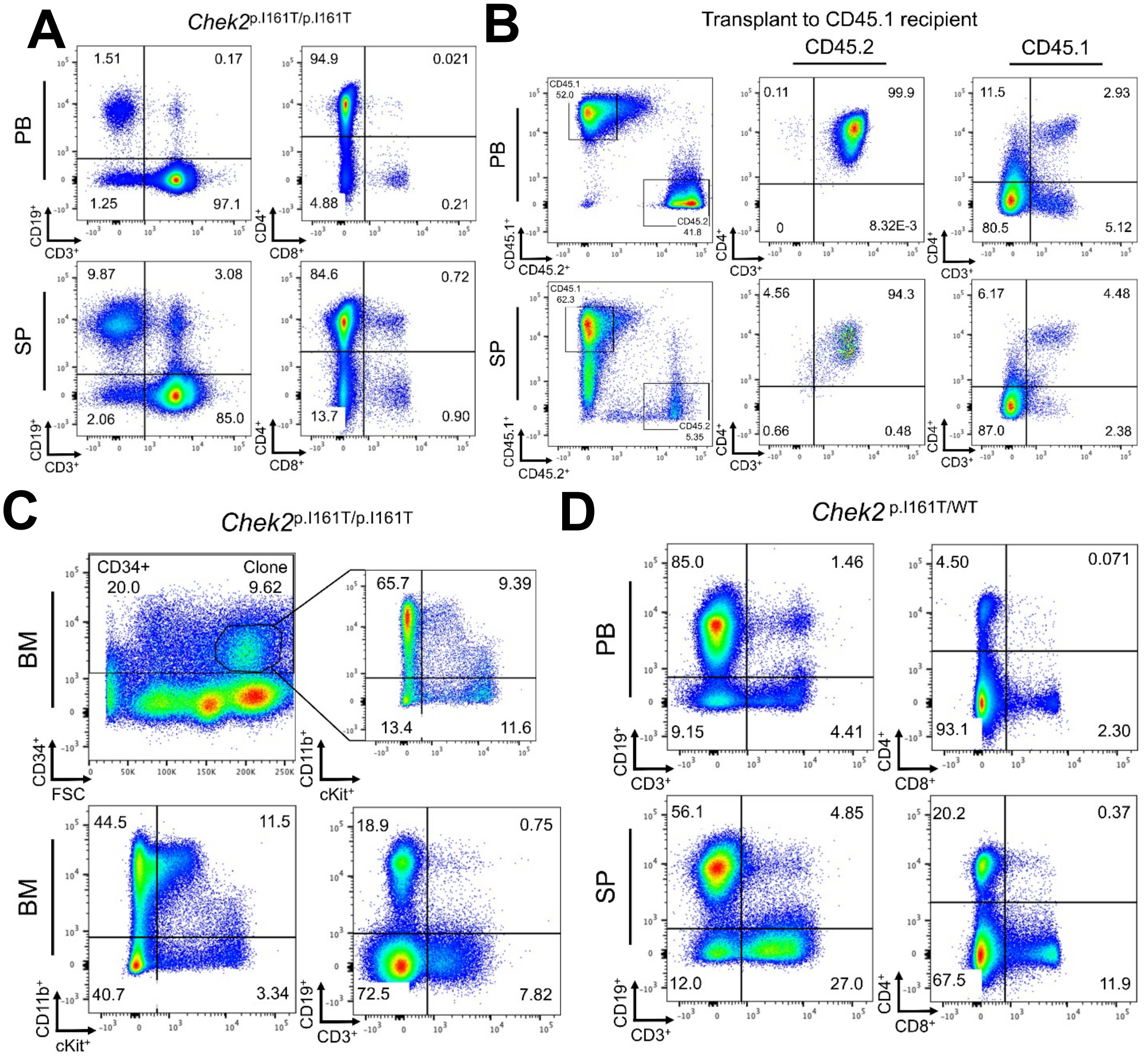

Supplemental Figure 16. Representative flow cytometry plots for endpoint *Chek2* mice. (A) Representative multicolor flow cytometry plot from a peripheral blood (PB) or spleen (SP) from a homozygous (*Chek2*$^{p.I161T/p.I161T}$) mouse that developed a CD3+CD4+ leukemia. (B) Representative multicolor flow cytometry plot from PB or SP from a CD45.1 recipient mouse transplanted with a CD3+CD4+ leukemia from a CD45.2 donor. (C) Representative flow cytometry plots from bone marrow (BM) for a homozygous (*Chek2*$^{p.I161T/p.I161T}$) mouse with evidence of a myeloid malignancy. (D) Representative flow cytometry plots from PB and SP for a heterozygous (*Chek2*$^{p.I161T/wt}$) mouse with a CD19+ B-cell proliferation.

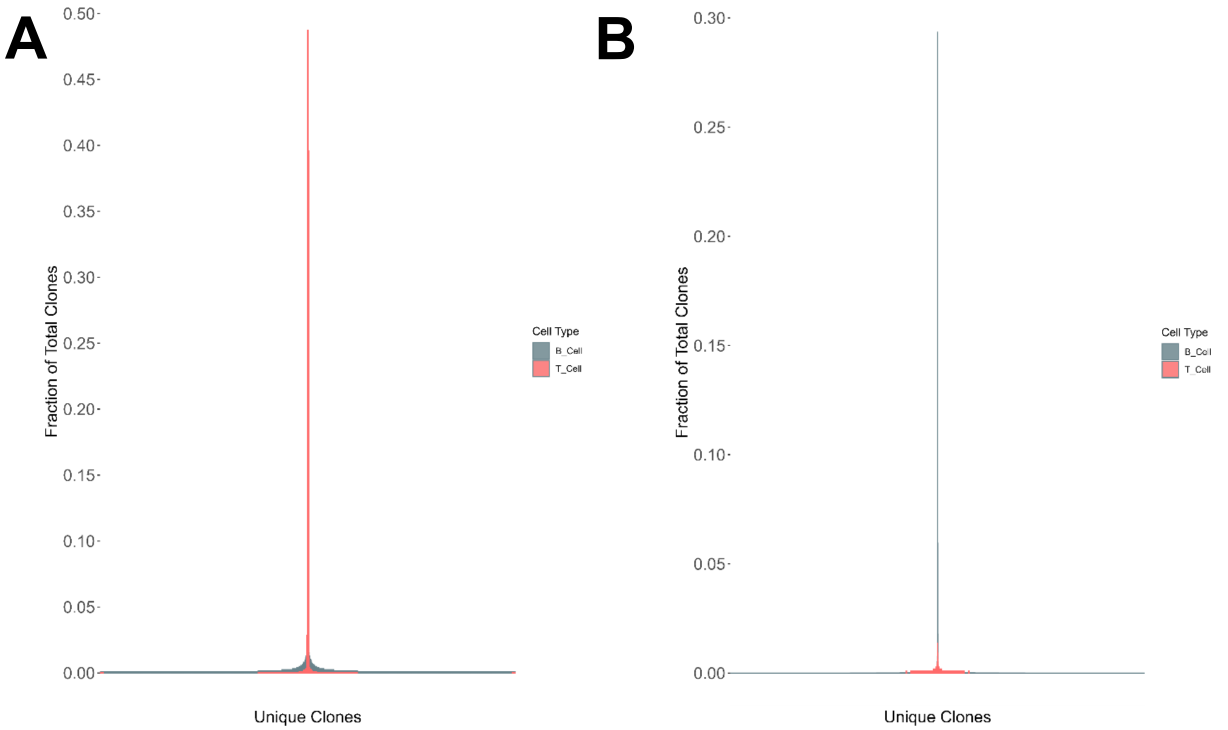

Supplemental Figure 17. Representative T-cell receptor (TCR) and B-cell receptor (BCR) sequencing and clonotype calling from *Chek2* endpoint mice. (A) T-cell receptor (TCR) sequencing and clonotype calling from a CD45.1 recipient mouse transplanted with a CD3+CD4+ leukemia from a CD45.2 donor. (B) B-cell receptor (BCR) sequencing and clonotype calling from a mouse with a CD19+ B-cell proliferation.
